# Novel GelMA/GelMA-AEMA Hydrogel Blend with Enhanced Printability as a Carrier for iPSC-Derived Chondrocytes In Vitro

**DOI:** 10.3390/gels11090698

**Published:** 2025-09-02

**Authors:** Paulo A. Amorim, Hannah Agten, Margaux Vermeulen, Sandra Van Vlierberghe, Liesbet Geris, Veerle Bloemen

**Affiliations:** 1 Department of Materials Engineering, Surface and Interface Engineered Materials (SIEM), Group T Leuven Campus, KU Leuven, 3000 Leuven, Belgium; 2 Skeletal Biology and Engineering Research Center, KU Leuven, 3000 Leuven, Belgium; liesbet.geris@kuleuven.be; 3 Prometheus, Division of Skeletal Tissue Engineering, Skeletal Biology and Engineering Research Center, KU Leuven, 3000 Leuven, Belgium; 4 BIO INX BV, 9052 Zwijnaarde, Belgium; 5 Polymer Chemistry & Biomaterials Group, Centre of Macromolecular Chemistry, Ghent University, 9000 Ghent, Belgium; 6 Biomechanics Section, Department of Mechanical Engineering, KU Leuven, 3001 Leuven, Belgium; 7 Biomechanics Research Unit, GIGA In Silico Medicine, University of Liège, 4000 Liège, Belgium

**Keywords:** GelMA, 3D bioprinting, rheology, IPSC, cartilage tissue engineering

## Abstract

Cartilage tissue engineering aims to restore damaged cartilage using biomaterials, cells, and/or biological cues to support cell growth and tissue repair. Although in the past decades scientific advances have moved the field forward, their translation to a clinical setting is still hampered. One major hurdle to take is to reduce process variability to ensure a predictable biological outcome. Using enabling technologies such as bioprinting has shown the potential to improve process robustness. However, developing bioinks that balance printability with biological functionality remains a major challenge. This study presents the development and structure–property relationships of a novel gelatin-based hydrogel blend, GelMA/GelMA-AEMA, optimized for extrusion-based bioprinting (EBB) while maintaining the crucial biological properties of GelMA for tissue engineering applications. The novel GelMA/GelMA-AEMA blend demonstrated superior flowability and printability compared to GelMA, effectively addressing common 3D-printing defects such as filament shape inhomogeneity. A systematic rheological characterization revealed that the blend exhibits a softer, elastically dominated structure with improved compliance. The blend behaves as a yield-stress fluid with a strong shear-thinning degree, making it highly suitable for EBB. The superior flow properties of the blend are deemed to enhance bond slippage and stress-induced orientation of its more imperfect gel structure, resulting in greater macroscopic deformation and enhanced print fidelity. In addition, histological assessment of a 21-day in vitro study with iPSC-derived chondrocytes suggested that the blend is at least equally performant as GelMA in supporting matrix formation. Histological analysis shows similar matrix deposition profiles, whereas gene expression analysis and compression tests even have suggested superior characteristics for cartilage TE. This study emphasizes the central role of rheology in bioink development and provides foundations for future material development for EBB, with potential implications for cartilage tissue engineering.

## 1. Introduction

Tissue engineering (TE) has evolved as an interdisciplinary field, making use of engineering strategies to address major medical challenges, such as tissue regeneration and the growing need for organ transplants [1]. Among the various materials utilized in TE, gelatin methacryloyl (GelMA) hydrogels have gained prominence due to their excellent biocompatibility and chemical similarity to the extracellular matrix of connective tissues [2,3]. Since their introduction in the early 2000s, GelMA hydrogels have become a gold-standard material, particularly in extrusion-based 3D bioprinting (EBB) [4,5,6]. EBB is a modality of the emerging 3D bioprinting technology, which is extensively employed in the field of biofabrication to enable the fabrication of complex, cell-laden structures with potential clinical applications, such as skin, bone, and cartilage tissue engineering and organ-on-a-chip models [7,8].

Despite their importance, GelMA hydrogels are not without limitations [9,10]. One key challenge is their tendency to exhibit filament shape inhomogeneity during the EBB process, a phenomenon similar to extrudate distortions observed in polymer melt extrusion [11,12,13]. This issue is detrimental to print fidelity and the integrity of printed structures. In addition, GelMA hydrogels have a narrow printing window that requires precise temperature control of both the printhead and substrate to avoid nozzle clogging due to premature gelation [14]. These printability-related challenges, along with batch-to-batch variability, are significant barriers to their clinical application [15]. In gelatin-derived materials, batch variability is largely due to the lack of standardized functionalization protocols, and variables such as differences in modification degree, molar mass, and gelatin source [16]. Moreover, the post-printing mechanical properties of GelMA are relatively low for applications in osteochondral tissue engineering and inorganic biofunctional fillers are often employed to enhance the elastic modulus of constructs and their osteoconductive and osteoinductive properties [17]. In addition to chemical and rheological modifications, recent studies have also explored the inclusion of nano-clay additives to improve water permeability and biofunctionality of hydrogel constructs, particularly in the context of tissue engineering [18]. Collectively, these challenges and strategies highlight the need and efforts for alternative hydrogel formulations that can improve printability while retaining the biological properties of GelMA, thereby overcoming some of its important limitations. In response to some of these challenges, especially the tendency of GelMA to exhibit extrudate distortions [13], our study introduces a novel hydrogel blend composed of GelMA and gelatin methacryloyl-aminoethyl-methacrylate (GelMA-AEMA).

The combination of these two gelatin derivatives is designed to enhance material performance in EBB by improving its flow properties, print fidelity, and mechanical compliance, without compromising the biological performance of GelMA. GelMA-AEMA, initially explored for two-photon 3D printing, does not form gels at room temperature up to concentrations of 15 *w*/*v*% [19], making it an interesting candidate for blending with GelMA to address elastic instabilities arising from the EBB process. Herein, we provide a comprehensive evaluation of the GelMA/GelMA-AEMA hydrogel blend, including its rheological properties, printability, and biological performance in the presence of induced-pluripotent stem cell (iPSC)-derived chondrocytes for cartilage tissue engineering applications. The introduction of this blend represents an advancement in the development of hydrogels tailored for EBB, paving the way for more effective and reliable EBB processes in tissue engineering.

## 2. Results

### 2.1. Bulk Shear Rheology

#### 2.1.1. Oscillatory Shear Measurements of Cell-Free and Cell-Laden Hydrogels

**Figure 1:**
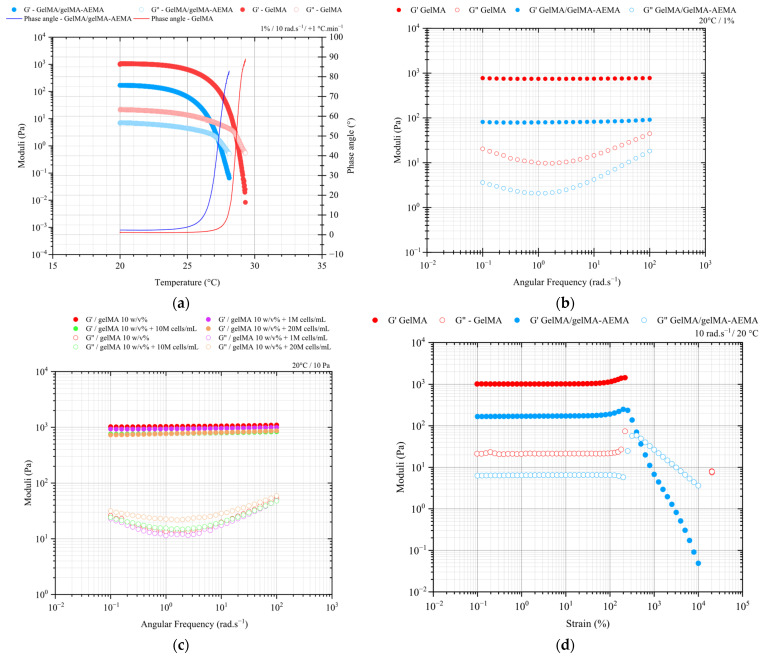
(**a**) Dynamic mechanical thermal behavior of GelMA (red circles) and GelMA-AEMA (blue circles) obtained via oscillatory shear from 20 to 40 °C at an angular frequency of 10 rad.s^−1^ and a ramp of +1 °C.min^−1^. (**b**) Mechanical spectra of cell-free hydrogels at 1% strain and 20 °C and (**c**) mechanical spectra of GelMA 10 *w*/*v*% physical hydrogel and GelMA 10 *w*/*v*%/hPDC at the cell densities of 0.1, 1, and 2×10^7^ cells per mL at 50 Pa and 20 °C. (**d**) Strain-amplitude sweep of GelMA 10 *w*/*v*% and GelMA/GelMA-AEMA at 1:1 *v*/*v* physical hydrogels at 10 rad.s^−1^ and 20 °C. Dynamic moduli corresponding to torques M > 0.10 μN.m and phase angles (δ) in the interval 0 < δ < 90° within the linear viscoelastic regime, as determined in the strain-sweep test in Figure 1c.

The temperature-dependent dynamic mechanical behavior of GelMA and GelMA/GelMA-AEMA physical hydrogels was investigated using small-amplitude oscillatory shear measurements, as shown in Figure 1. The results of the rheological characterization of GelMA-AEMA were not incorporated due to its very low dynamic moduli at 20 °C, which led to inaccurate data due to torque values approaching the threshold resolution of the instrument and inertial artifacts.

In Figure 1a, at 20 °C, both materials exhibited solid-like behavior, with the storage modulus predominating over the loss modulus. As temperature increases, the moduli decline abruptly, indicating a sharp transition from solid- to liquid-like behavior around 29 °C for GelMA and 27 °C for GelMA/GelMA-AEMA.

The linear viscoelastic moduli as a function of the angular frequency, as depicted in Figure 1b, revealed that both systems exhibit a gel character, evidenced by the dominance of the storage modulus (G′) over the loss modulus (G″) across four orders of magnitude of angular frequencies. Additionally, the blend hydrogel is a softer solid, given the closer proximity between the storage and loss moduli in comparison to neat GelMA. Also displayed in Figure 1b, the GelMA/GelMA-AEMA physical hydrogel showed a lower dynamic moduli than GelMA. At mid to high frequencies, the more pronounced frequency-dependence of the loss modulus of the blend suggests that a higher degree energy dissipation in the network structure is present at shorter length scales of its structure. The addition of human periosteum-derived cells to GelMA did not substantially alter its rheological behavior, making it just slightly softer, as seen in Figure 1c. This suggests that even at cell densities as high as 2.10^7^ cells/mL, GelMA bioinks retained their strong gel character, given by their frequency-independent spectra. These results corroborate previous research addressing the effect of high cell densities on the rheology of GelMA 10 *w*/*w*% [20,21].

In the strain-amplitude sweep experiments of Figure 1d, both GelMA and GelMA/GelMA-AEMA hydrogels revealed a broad linear viscoelastic region (LVR), with reversible deformations occurring up to very high strain levels. However, beyond this regime, strain-hardening and strain-softening were particularly observed for the GelMA/GelMA-AEMA system, which transitioned from solid- to liquid-like behavior at strains between 300% and 400%, indicating the onset of structural breakdown [22,23].

#### 2.1.2. Shear Creep Measurements and Determination of the Burgers Viscoelastic Model Parameters

**Figure 2:**
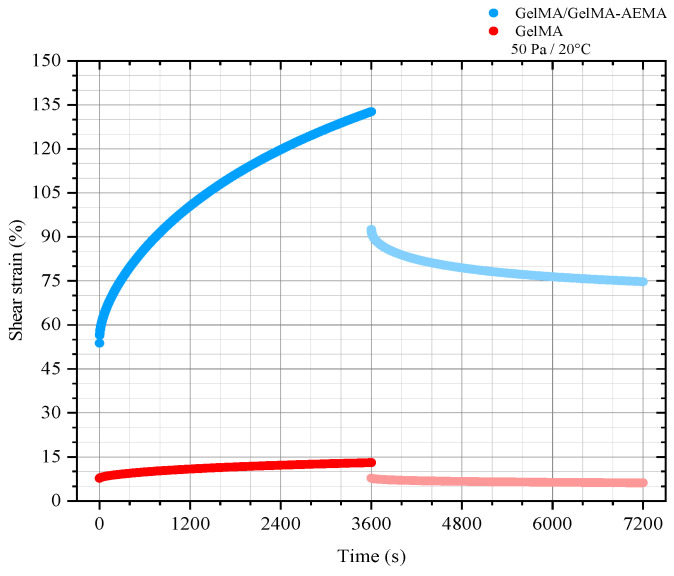
Shear strain functions of time for a constant shear stress of 50 Pa, within the linear viscoelasticity regime, for GelMA and GelMA/GelMA-AEMA physical hydrogels.

The shear strain as a function of time at the nominal constant shear stress of 50 Pa applied to GelMA and GelMA/GelMA-AEMA is presented in Figure 2. During the creep stage, both physical hydrogels reached stationary shear rates, suggesting flow [24]. The GelMA/GelMA-AEMA blend hydrogel demonstrated a creep behavior more akin to a liquid, whereas GelMA appeared to be approaching an equilibrium, suggesting a more solid-like character. However, residual deformation observed during the recovery stage suggests that GelMA must also be classified as a viscoelastic liquid within the experimental time scale. A significant difference in the strain levels achieved by the two materials is observed. The blend hydrogel deformed nearly an order of magnitude more than GelMA under the same applied stress level, indicating that the blend hydrogel can achieve larger deformation levels more quickly than GelMA, characterizing its more compliant behavior.

Due to their viscoelastic liquid behavior at these time scales of experimentation, the creep function for the Burgers’ model, a four-element mechanical model of a viscoelastic liquid (Equation (1)), was fitted to the data to quantify the main retardation times governing the behavior of GelMA and GelMA/GelMA-AEMA physical gels. The exponential regression results are shown in Figure S1. The analyzed data correspond to the creep stage under a shear stress of 50 Pa. The choice of the mechanical model shows excellent agreement with the creep data for both GelMA and the GelMA/GelMA-AEMA blend hydrogel. This is given by the adjusted coefficients of determination, which are higher than 0.99 for both systems (Figure S1). Material parameters of the Maxwell and Kelvin–Voigt elements used in the model were extracted from the data, and the zero-shear viscosities of the Maxwell element (η_M_) were obtained from the linear regression of the steady-state region of the creep curve. These parameters and the derived retardation times are presented in Table 1.

#### 2.1.3. Steady-State Rotational Measurements and the Determination of the Herschel–Bulkley Model Parameters

**Figure 3:**
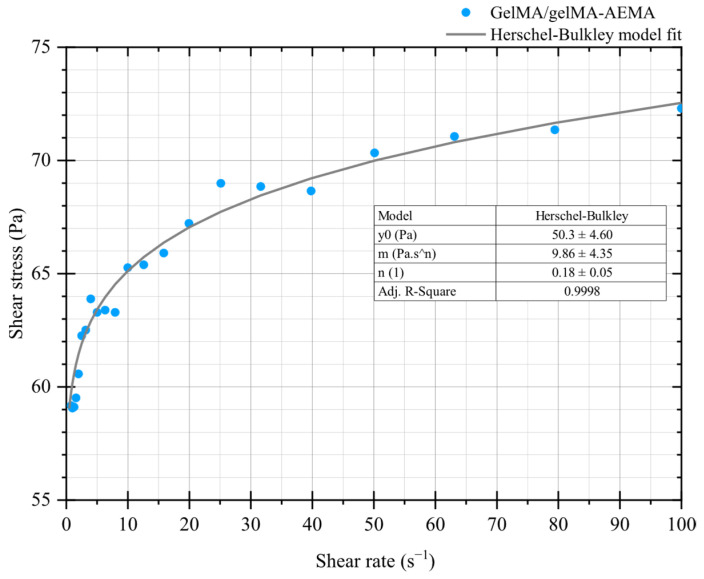
Flow curve of GelMA/GelMA-AEMA and adjustment of the Herschel–Bulkley fluid model.

GelMA at 10 *w*/*v*% was not sufficiently soft and malleable for flow curve experiments, as can be observed from the previous results. GelMA shows brittle fracture upon large strains, contrasting with the GelMA/GelMA-AEMA blend (Figure 1d). Therefore, only the novel blend was investigated in the framework of steady-state rotational measurements for presence of yield stress. The yield stress was determined using the Herschel–Bulkley model, which provided a value of yield stress of 50.3 ± 4.60 Pa, a consistency index of 9.86 ± 4.35 Pa·s^n^, and a flow index of 0.18 ± 0.05, corresponding to a high shear-thinning behavior. The coefficient of determination (R^2^ = 0.99) indicates great agreement, and these results are shown in Figure 3.

### 2.2. 3D-Printing Validation

**Figure 4:**
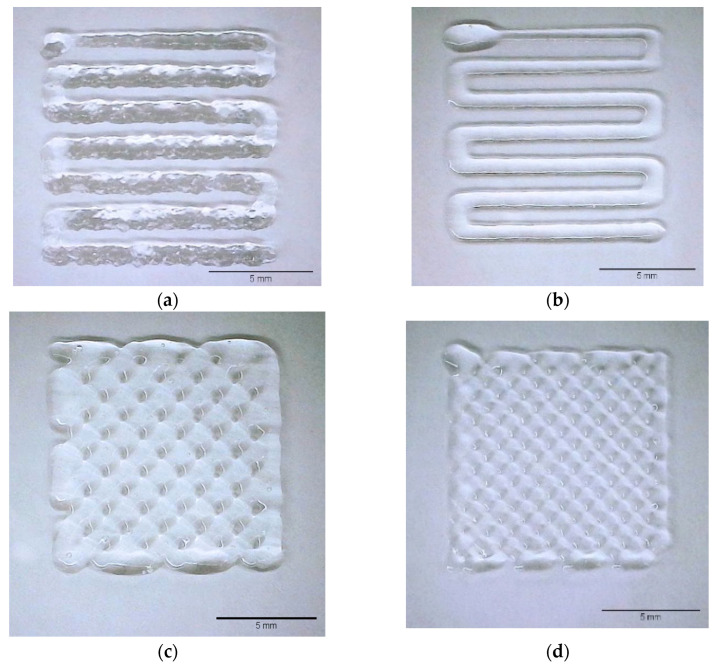
3D-printing validation and qualitative superficial aspect comparison: (**a**) the state-of-the-art material GelMA 10 *w*/*v*% and (**b**) the novel GelMA/GelMA-AEMA hydrogel blend. Printing parameters: T = 20 ± 2 °C, tapered nozzle of 0.250 mm diameter, v_P_ = 10 mm.s^−1^, (**a**) 3 μL.s^−1^ and (**b**) 1 μL.s^−1^. These flow rate values were obtained empirically and qualitatively as the flow rate at which filaments are formed for each material. Optimization of the printing speed to match the average fluid velocity according to the preset volumetric flow rate of 1 μL.s^−1^: (**c**) 10 mm.s^−1^ and (**d**) 20 mm.s^−1^ conducted at the room temperature of 20 ± 2 °C using a tapered nozzle of 0.250 mm for GelMA/GelMA-AEMA.

3D-printing validation of the novel GelMA/GelMA-AEMA hydrogel blend was conducted and compared qualitatively with GelMA 10 *w*/*v*%, as shown in Figure 4a,b. Figure 4a,b present the results of empirical filament formation tests conducted at constant volumetric flow rates for GelMA and the GelMA/GelMA-AEMA blend, respectively. These experiments aimed to identify the minimum extrusion flow rates that ensured continuous and stable filament formation without nozzle clogging or filament breakage. The threshold volumetric flow rates were empirically determined to be 3 μL/s for GelMA and 1 μL/s for the GelMA/GelMA-AEMA blend.

To further develop these empirical observations and define optimal printing conditions, a macroscopic mass balance (Equation (4)) was applied using the known nozzle diameter (*d* = 0.250 mm) and the empirically determined flow rate for the blend (*Q* = 1 μL/s). This resulted in an optimal printhead translation speed of 20 mm.s^−1^. Figure 4c,d illustrate the application and validation of this approach, where the calculated speed produced homogeneous and continuous filaments during printing of the GelMA/GelMA-AEMA blend.

The 3D-printed filaments of GelMA, shown in Figure 4a, displayed a rough morphology similar to extrudate distortions, whereas those of the GelMA/GelMA-AEMA blend, shown in Figure 4b, were smooth. This suggests that the GelMA/GelMA-AEMA blend flowed more consistently, effectively copying the shape of the printing die, in contrast to GelMA, which exhibited signs of extrusion instability akin to elastic instabilities observed for polymer melts at high shear rates.

We demonstrated the printability potential of the GelMA/GelMA-AEMA blend at two different printing speeds: 10 mm.s^−1^ and the optimized printing speed of 20 mm.s^−1^ given by Equation (4). The hydrogel blend maintained good printability at both speeds, producing well-defined, uniformly spaced filaments.

### 2.3. Biological Evaluation of Encapsulated iPSC-Derived Chondrocytes

**Figure 5:**
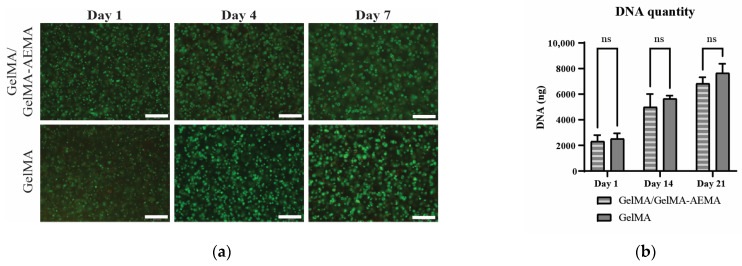

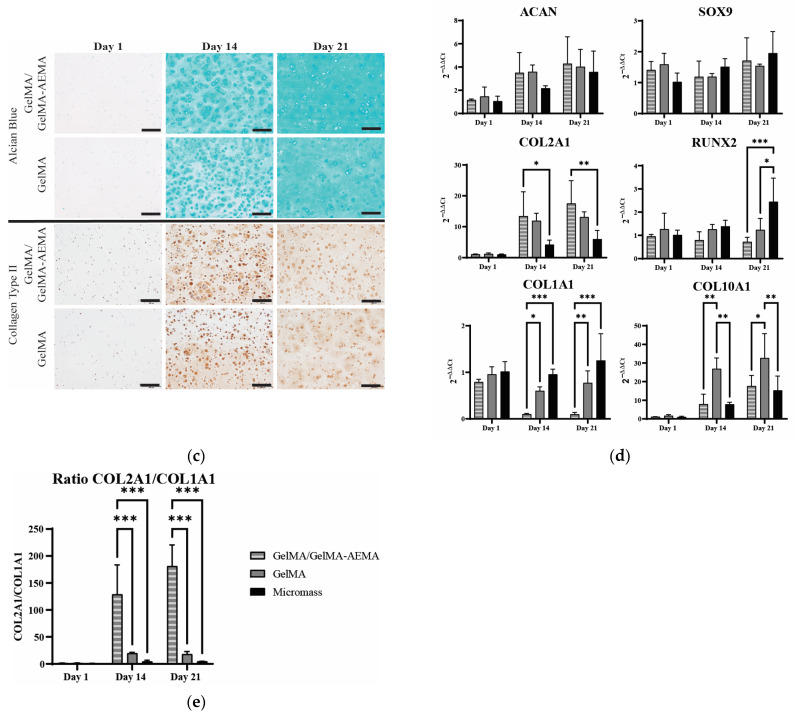
Biological evaluation of encapsulated iPSC-derived chondrocytes in GelMA/GelMA-AEMA blend versus GelMA hydrogel. (**a**) LIVE/DEAD staining on days 1, 4, and 7 after construct fabrication. Scale bar: 200 µm. (**b**) DNA quantification of cell-laden on day 1, 14, and 21 after construct fabrication plotted as mean ± standard deviation (*n* = 3). Unpaired *t*-test with Welch correction per time point; individual variances were computed for each comparison, and no significant differences were detected (*p* < 0.05). (**c**) Alcian Blue staining for glycosaminoglycans (top) and collagen type II immunohistochemistry (bottom) of iPSC-derived chondrocyte-laden GelMA/GelMA-AEMA hydrogel blend and GelMA hydrogel. Scale bar: 200 µm. (**d**) Gene expression analysis of iPSC-derived chondrocytes encapsulated in GelMA/GelMA-AEMA hydrogel blend and GelMA hydrogel versus cells only (micro-masses). Relative mRNA expression of cell-laden hydrogels (*n* = 3), represented with 2^−ΔΔCt^ method [20], with HPRT1 as housekeeping gene and normalized to gel-free conditions (micro-masses) on day 1. (**e**) Ratio of COL2A1 to COL1A1 expression of iPSC-derived chondrocytes encapsulated in GelMA/GelMA-AEMA blend and GelMA compared to gel-free conditions (micro-mass). Statistical analysis consists of 2-way ANOVA followed by Tukey’s multiple comparisons test with * *p* < 0.05, ** *p* < 0.01, *** *p* < 0.001.

LIVE/DEAD staining on cast and UV-crosslinked constructs of iPSC-derived chondrocytes in GelMA/GelMA-AEMA blend and GelMA samples (Figure 5a) showed that the majority of cells survived the construct fabrication procedure and confirmed that high viability was obtained after 1 week of in vitro culture, and to a similar extent as the cells encapsulated in GelMA. When comparing DNA quantities in both cell-laden hydrogels at each time point (Figure 5b), no significant differences were observed. In both matrices, DNA quantity increased about 2-fold by day 14 and about 3-fold by day 21, compared to day 1.

Figure 5c shows that iPSC-derived chondrocytes were able to deposit glycosaminoglycans into the hydrogel matrix. While no positive Alcian Blue staining was observed on day 1 of in vitro culture, an intense staining was seen on day 14 that is most intense in the pericellular space. By day 21 of in vitro culture, the staining also intensified in the intercellular space. Qualitatively, no differences were observed between the GelMA hydrogel and the novel blend. Immunohistochemistry staining showed that iPSC-derived chondrocytes in the GelMA/GelMA-AEMA hydrogel blend deposit human collagen type II, the most abundant structural protein in cartilage matrix. At day 14, the immunostaining was strongest pericellularly, while the protein was more dispersed throughout the matrix by day 21. This deposition pattern was very similar to iPSC-derived chondrocytes in the GelMA hydrogel. When comparing Alcian Blue and collagen type II staining in hydrogel-based constructs to the gel-free micro-mass controls (Supplementary Information, Figure S3), a similar staining intensity was observed, although a lower cell number per area is present in the hydrogel conditions. Collagen type I was not detected throughout the 21-day in vitro culture period (Supplementary Information, Figure S4).

The positive staining for Alcian Blue and collagen type II by iPSC-derived chondrocytes was also confirmed in bioprinted GelMA/GelMA-AEMA samples (Supplementary Information, Figure S6).

Gene expression analysis (Figure 5d,e) was used to quantitatively compare the gene-specific mRNA quantification of cells encapsulated in the novel blend to benchmark hydrogel GelMA and to cells cultured in the absence of gel, in micro-mass culture. Gene expression was normalized to the expression on day 1 in gel-free conditions. The expression of chondrogenic transcription factor SOX9 was not significantly different between the various conditions. ACAN, a gene coding for the main proteoglycan seen in cartilage tissue, also did not show significant differences between gel-free and both hydrogel conditions, confirming what was observed in the stainings for glycosaminoglycans. Interestingly, the novel blend showed significantly lower expression of COL1A1 (10-fold downregulation compared to 1.3-fold for GelMA) and significantly higher expression of COL2A1, compared to gel-free controls. These differences in collagen-related gene expression yield a COL2A1/COL1A1 ratio of 181.2 ± 39.3 at day 21, compared to 18.1 ± 4.96 for GelMA and 4.74 ± 0.23 for gel-free conditions (Figure 5e). A milder upregulation in hypertrophic marker COL10A1 (17-fold) compared to GelMA (32-fold) was observed and the early osteogenic marker RUNX2 showed no upregulation, in contrast to gel-free conditions.

### 2.4. Mechanical Properties of UV-Cured Constructs

**Figure 6:**
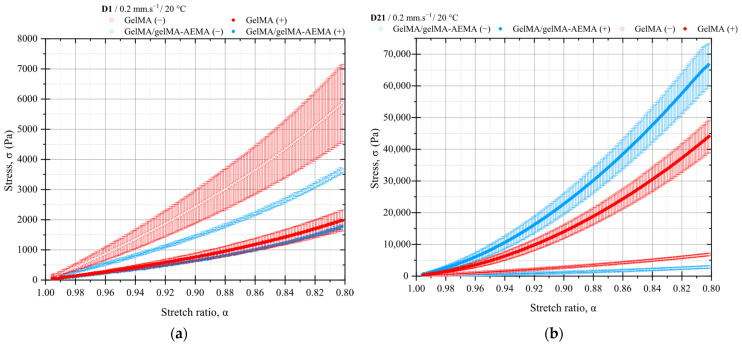
Compression testing of GelMA and GelMA/GelMA-AEMA chemical hydrogels and cell-laden constructs at (**a**) day 1 and (**b**) day 21. Measurements were performed at 0.2 mm.s^−1^ and 25 °C, with a maximum strain of 20% of the total thickness (2 mm) to avoid interference with bottom plate. Hybrid hydrogel specimens with dimensions of 4 × 4 × 2 mm (W × L × D) were tested. The symbols (+) and (−) designate cell-laden and cell-free control samples, respectively. Error bars correspond to the standard deviation of the mean.

The results of the compression tests of UV-cured GelMA and GelMA/GelMA-AEMA constructs are presented in Figure 6. All the results of the statistical analysis are presented in Tables S2 and S3 of the Supplementary Information. The curve-fitting parameters of the thermodynamical equation of state of statistical rubber elasticity can be found in Figure S7 of the Supplementary Information.

#### 2.4.1. Mechanical Testing of Cell-Free Constructs

The mechanical properties of GelMA and GelMA/GelMA-AEMA chemically crosslinked hydrogels, both at the concentration of 10 *w*/*w*% and in presence of 6 mM of lithium phenyl-2,4,6-trimethylbenzoylphosphinate (LAP, CAS 85073-19-4) photoinitiator, were first studied in the absence of cells after 1 and 21 days in a differentiation medium, as shown in Figure 6a and Figure 6b, respectively. The results reveal that GelMA exhibited a higher initial modulus of elasticity compared to the GelMA/GelMA-AEMA blend. At day 1, the elastic modulus of GelMA was 7302.13 ± 22.03 Pa, which increased slightly to 8455.41 ± 19.29 Pa by day 21. In contrast, the GelMA/GelMA-AEMA blend had a lower initial modulus of 4406.83 ± 14.05 Pa at day 1, which decreased to 3489.50 ± 12.51 Pa by day 21. These changes suggest that GelMA undergoes further mechanical strengthening during the culturing media, while the blend hydrogel may experience some degree of structural degradation or reorganization in the same period, in the absence of cells.

#### 2.4.2. Mechanical Testing of Cell-Laden Constructs

Significant differences were observed in the compressive strength of cell-laden constructs over the 21-day culture period, as depicted in Figure 6b. Both GelMA and GelMA/GelMA-AEMA hydrogels demonstrated a substantial increase in the modulus of elasticity, K, in the presence of cells. For GelMA, the modulus K increased from 2334.72 ± 10.48 Pa at day 1 to 41,050.61 ± 812.11 Pa at day 21, indicating an approximately 18-fold increase. The GelMA/GelMA-AEMA blend showed an even more pronounced increase, with the modulus K increasing from 2010.84 ± 9.99 Pa at day 1 to 67,687.55 ± 1081.36 Pa at day 21, corresponding to a 34-fold increase in compressive strength.

#### 2.4.3. Comparative Analysis Between Materials

At day 21, the comparison between the cell-laden constructs of GelMA and GelMA/GelMA-AEMA showed a substantial difference in their compressive strength. The GelMA/GelMA-AEMA constructs were significantly stiffer, as it can be inferred from Figure 6b, with a mean modulus of elasticity of 67,687.55 ± 1081.36 Pa, compared to 41,050.61 ± 812.11 Pa for GelMA. The statistical analysis confirmed that these differences were significant at the 0.01 level.

#### 2.4.4. Structural Evolution During Culture

The evolution of the hydrogel structures during the 21-day in vitro culture suggests that cell activity plays a crucial role in modulating the mechanical properties of the systems. The cell-laden GelMA/GelMA-AEMA constructs demonstrated a remarkable increase in modulus, potentially due to enhanced crosslinking or network reinforcement driven by cellular processes, such as production of extracellular matrix. This phenomenon was more pronounced in the GelMA/GelMA-AEMA blend, leading to a more mechanically robust construct compared to GelMA.

## 3. Discussion

Oscillatory shear measurements provided valuable insights into the rheological behavior of the GelMA and GelMA/GelMA-AEMA hydrogels. The observed temperature-dependent transition from solid- to liquid-like behavior and the differences in denaturation activation energies between the two materials highlight the influence of mechanical blending on the resulting blend hydrogel structure. The difference of approximately 2 °C may be attributed to phenomena such as steric hindrance of bulkier side groups and/or shorter chains of GelMA-AEMA, preventing hydrogen bond formation, and/or a slightly more polydisperse and lower distribution of denaturation activation energy in the blend hydrogel compared to GelMA. However, an in-depth investigation is out of the scope of this research. In terms of the materials performance in EBB, the very abrupt decrease in the dynamic moduli of the gels with temperature makes it challenging to tailor their viscoelastic properties by means of temperature control in printing conditions.

Both materials show mechanical spectra of stable gel networks within the timescales probed in our frequency sweep experiments. The softer 3D network of the blend hydrogel, as evidenced by its mechanical spectrum, suggests a more malleable structure, which is advantageous for EBB [25]. Furthermore, the frequency sweep experiments confirmed that the GelMA/GelMA-AEMA hydrogel forms a more non-affine network structure than GelMA [26], leading to a larger mesh size of the physical gel and, consequently, its lower storage modulus and higher loss tangent values. These imperfections can be attributed, for instance, to entanglements, dangling, and loop structures, as well as large-cluster reorganization and sol phase embedded in the 3D network, and contribute to the physical blend hydrogel increased deformability [26], making it more suitable for EBB. The strain-amplitude sweep experiments further supported the superior suitability of the GelMA/GelMA-AEMA hydrogel for EBB. The blend exhibited strain-hardening followed by strain-softening behavior, which interestingly is typical of associative systems such as telechelic polymers [27]. Similar transitioning capability was not observed for GelMA, which fractures in a brittle manner, hence suggesting that the novel gelatin blend is more malleable and adequate for extrusion-based 3D bioprinting due to its higher ability to flow upon large strains. Our type B GelMA, on the other hand, showed rheological behavior similar to that previously described in the literature [28], fracturing in a brittle mode. Gelatin supramolecular networks are primarily stabilized by hydrogen bonds between amine and carboxylic acid groups in proline and hydroxyproline residues, forming junction zones [29,30]. Pristine gelatin typically exhibits a remarkable elastic-dominated response up to brittle failure at strain levels of 200–300%, regardless of polymer concentration [31,32]. Our results indicate that despite reduced hydrogen bonding in GelMA due to methacryloyl modification, its large-strain rheological behavior remained quite similar to that of pristine gelatin. This can be attributed to the few methacryloyl groups per chain. In addition, deviation from affine entropic elasticity in gelatin is attributed to structural inhomogeneities and entanglements that do not participate in the supramolecular gel structure [33].

Large deformations likely lead to the ‘unzipping’ of triple helices, making fracture strain or stress rate-dependent [22,23]. Our findings suggest that deviation from entropic elasticity and bond slippage may be facilitated in the GelMA/GelMA-AEMA blend, even at high rates such as 10 rad.s^−1^. This can once again be attributed to weakened hydrogen bonds from the flexible coils and bulky side groups of GelMA-AEMA, resulting in a more imperfect and, thus, more malleable hydrogel network [19]. Also, reorganization of large-scale structure clusters under shear may contribute to this observation. Overall, the oscillatory tests suggest that the blend hydrogel offers a better balance of viscous and elastic properties compared to the current gold-standard GelMA for the development of bioinks for EBB. However, further understanding of the dual viscoelastic nature of these materials requires long-term tests, such as creep tests.

The results of creep measurements can be explained by the known properties of gelatin [32]. Over longer times, only the junction zones and permanently trapped entanglements support stress, as described by Ross-Murphy [31]. This fact explains the terminal behavior observed for both GelMA and GelMA/GelMA-AEMA. The key difference between them lies in the rate at which the rupture and reformation of junction zones occur, leading to the different creep rates observed. The results suggest that the junction zones in GelMA/GelMA-AEMA break faster than in GelMA, corroborating the results of oscillatory measurements. This phenomenon can be attributed to the further functionalization reaction of the carboxylic acid groups in GelMA chains, which results in GelMA-AEMA. This additional functionalization likely leads to less hydrogen bonds, preventing folding of the polymer chains in triple helices, as indicated by our systematic rheological characterization. Additionally, the dangling structures and entanglements present in the blend structure may contribute to its more pronounced relaxation. From an engineering perspective, the GelMA/GelMA-AEMA physical hydrogel blend can be considered a self-healing biomaterial. The blend exhibits self-healing behavior because at small deformations (as shown in the frequency sweep test), it behaves as a viscoelastic solid, but upon straining, the blend transitions from a solid to a liquid regime. This transition was also observed during the 3D-printing validation, in which the material deposited onto the substrate could be more compactly stacked. The blend transitions to a liquid-like regime under large strains inside the cartridge and through the nozzle but returns to a solid-like regime afterwards. The flow behavior observed in the creep tests, coupled with the rapid elastic recovery of the blend, further supports its self-healing character.

The main retardation times derived from the Burgers’ creep curve adjustment are similar for both GelMA and GelMA/GelMA-AEMA physical hydrogels. These retardation times correspond to the delay caused by the Kelvin–Voigt element’s dashpot in the otherwise instantaneous stretching of its spring under loading. Although this parameter does not reveal significant differences in the dynamics of the two materials, chain dynamics of gelatin are governed by two distinct characteristic times. A faster relaxation mode occurs at shorter times, related to the elastic active network chains and their mesh size, while a slower relaxation mode predicts the lifetime of the physical crosslinks, controlled by the slippage of these crosslinks [32]. Despite similar retardation times, the materials exhibit substantial differences in the dissipation behavior of the Kelvin–Voigt element. The approximately 19-fold decrease in the viscous component (η_k_) for the blend compared to GelMA suggests milder dissipative mechanisms in the renatured gelatin segments of the GelMA/GelMA-AEMA blend, aligning with our previous rheological observations. The analysis suggests that the sol fraction trapped within the gel network, resulting from spinodal decomposition, along with network imperfections such as dangling chains, entanglements, and large-scale cluster reorganizations, contributes to the mechanical response described by the Maxwell element. In contrast, the helical structures are primarily responsible for the mechanical behavior associated with the Kelvin–Voigt element. The parameters in Table 1 suggest that the junction zones in the blend hydrogel differ significantly from those in GelMA in terms of supporting mechanical loading, resulting in a more compliant, malleable GelMA/GelMA-AEMA blend.

Moving from a fundamental to a practical processing perspective, steady-state rotational measurements and the Herschel–Bulkley model can be useful to provide insightful information on the actual performance of our novel blend hydrogel under processing conditions. A strong shear-thinning behavior has been considered a cornerstone in the development of bioinks [34,35]. Several studies have shown correlations between strong shear-thinning behavior with increased cell viability and proliferation in tissue-engineered constructs [36,37,38]. The pronounced shear-thinning behavior, quantified by the low flow index *n*, implies that the material experiences a significant drop in viscosity under increasing shear rates, such as those encountered during extrusion through a nozzle. This facilitates smooth flow during deposition and rapid viscosity recovery once the shear is removed, which is essential for maintaining printed filament geometry and ensuring shape fidelity. The presence of a yield stress also implies the material remains stationary under low-stress conditions post-deposition, preventing undesirable spreading or sagging of printed structures. Together, these rheological characteristics not only enhance printability but also align with the requirements for high-resolution, self-supporting biofabrication systems where mechanical integrity and structural precision are important.

Our study has shown that the GelMA/GelMA-AEMA blend is a Herschel–Bulkley fluid displaying yield stress and an extreme shear-thinning behavior (*n* ≈ 0.2). Thus, another practical implication of a yield stress was applied to estimate the maximum attainable theoretical height of a hypothetical GelMA/GelMA-AEMA construct. The Von Mises criterion [39] and the relationship in Equation (3) predict a maximum attainable theoretical height of 9 mm. For constructs of which the heights are situated above this value, yielding occurs at the infinitesimal thin film at the bottom of the first layer withstanding all the printed structure above it. This means that above the critical height derived from the yield-stress threshold, shape fidelity is impaired and eventually lost. In addition, this estimation does not take into consideration possible shortcomings related to the fabrication process as well as the different devices and technologies commercially available. Nevertheless, the size of a cartilage patch needed in a critically sized joint defect is estimated to be in the range of 3 to 5 mm [40], which is substantially smaller than the theoretical estimate given by the Von Mises yield criterion for the hydrogel blend.

To provide visual evidence of the striking differences in terms of rheological behavior discussed so far, 3D-printing validation was conducted. The difference between GelMA and GelMA/GelMA-AEMA 3D-printed filaments depicted by Figure 4a and Figure 4b, respectively, is striking. The qualitative comparison between GelMA and the GelMA/GelMA-AEMA blend highlights the superior flow characteristics of the blend during 3D printing. The smooth morphology of the GelMA/GelMA-AEMA filaments indicates absence of flow instability, which is often seen in materials like GelMA that exhibit a slip-stick effect. This instability is linked to the inability of the material to relax stresses quickly enough during fast processing (high deformation rates), leading to extrusion defects that precede the fracture of the material at higher stresses. These flow instabilities occur when the time scales of the process are too fast so that the material is not able to relax the applied stresses [41]. In addition, it is worth noting that Equation (4) provides an approximation of the average flow velocity at the nozzle tip and does not account for the extrudate swell (Barus effect), which would reduce the actual extrusion speed. Thus, the printed filament is likely stretched during deposition. Remarkably, the hydrogel blend filaments remained stable under these conditions, suggesting that the applied printing speed compensates for this effect.

The successful 3D printing of the GelMA/GelMA-AEMA blend at both tested speeds confirms its suitability for extrusion-based 3D bioprinting. The consistent filament quality at different speeds suggests that the physical blend hydrogel can be processed under a variety of conditions without compromising printability and can be adjusted to ensure shape fidelity.

As our blend poses as a prominent candidate for tissue engineering using EBB, its biological performance was assessed by encapsulating iPSC-derived chondrocytes and assessing their behavior over a 21-day in vitro culture period within photocrosslinked hydrogels from the sol state at 37 °C. The photocrosslinking experiment described and presented in the Supplementary Information, Figure S2, was employed to estimate the initial mechanical properties of GelMA and GelMA/GelMA-AEMA hydrogels. As expected, the crosslinking reaction kinetics showed a faster rate of increase and stabilization of G’ for the GelMA/GelMA-AEMA blend compared to GelMA. This behavior can be attributed to the higher number of photo-sensitive moieties in the GelMA-AEMA due to its greater functionalization. Since the same photoinitiator concentration (i.e., 6 mM) was used for both hydrogels, the difference in plateau modulus convergence times (Figure S2) may be attributed to oxygen inhibition, which likely had a less pronounced effect on the blend hydrogel due to its higher number of available functionalities to react.

Starting from similar mechanical properties and mesh sizes, our first biological experiment revealed that the new hydrogel blend was able sustain high cell viability levels as the gold-standard GelMA within these experimental conditions. Hence, based on the viability and proliferation data, the addition of the aminoethyl methacrylate (AEMA) group did not significantly influence the inherent biocompatibility of GelMA, as it had been previously shown in 2D culture with murine fibroblasts and osteoblasts [19]. Also, this corroborates our finding that mesh size of the chemical blend hydrogel was not significantly impacted.

Deposition of cartilage-like matrix by chondrocytes encapsulated in the GelMA hydrogel was previously observed in in vitro culture periods of up to 8 weeks [9,42,43,44,45,46]. Our group has previously shown that chondrogenic matrix proteins were expressed after 21 days of in vitro culture and we observed a superior matrix-forming capacity by iPSC-derived chondrocytes compared to primary chondrocytes, possibly due to their younger age, as was described in pellet culture systems [47,48,49,50]. Nevertheless, the difference in collagen gene expression profiles between GelMA and the GelMA/GelMA-AEMA blend is strikingly pronounced. The novel blend caused stronger downregulation of COL1A1 compared to GelMA and a milder upregulation in COL10A1 compared to GelMA, suggesting a more stable cartilage phenotype. This was confirmed by a 10-fold higher redifferentiation index, given by the COL2A1/COL1A1 ratio, found in the presence of GelMA/GelMA-AEMA in comparison to GelMA. Other studies have investigated the influence of mechanical properties, such as matrix stiffness on chondrocyte differentiation and matrix production [46]. Bachmann et al. reported that hydrogels with elasticities matching those of the perichondral space (Young’s modulus, E = 30 kPA) have superior chondrogenic properties [51]. It has also been shown that softer hydrogels allow more collagen type 2 production [52].

UV-cured GelMA and GelMA/GelMA-AEMA prepared as described in the methods section were evaluated in terms of their mechanical properties in uniaxial compression tests and the neo-Hookean model. The experiment provided valuable insights into the mechanical performance and structural evolution of GelMA and GelMA/GelMA-AEMA hydrogels during a 21-day in vitro culture. The results indicate that the GelMA/GelMA-AEMA blend exhibits superior mechanical properties compared to GelMA, particularly in the presence of iPSCs. This finding suggests that the novel hydrogel blend not only maintains its structural integrity over time but also undergoes significant structural modifications due to cellular activity. The observed increase in modulus for both materials highlight the potential of these hydrogels for cartilage tissue engineering applications, where mechanical strength and resilience are critical for the successful integration of engineered constructs. The GelMA/GelMA-AEMA blend, with its enhanced mechanical properties, cell–material interactions and higher redifferentiation index represents a promising candidate for further studies aimed at developing cartilage tissue-engineered constructs.

## 4. Conclusions

In this study, we developed and characterized a novel photocurable gelatin-based hydrogel blend, GelMA/GelMA-AEMA, optimized for extrusion-based bioprinting. The blend is composed by GelMA and GelMA-AEMA at the volume fraction of 1:1, both components at the concentration of 10 *w*/*w*%. This blend retains the key biofunctional properties of GelMA while significantly improving flowability, printability, and shape fidelity compared to the current gold standard, GelMA 10 *w*/*w*%. Our systematic rheological characterization demonstrated that the novel blend behaves as a softer, highly compliant, yield-stress material governed by the Herschel–Bulkley model, which are characteristics that contribute to superior extrusion performance of the blend against GelMA.

Biologically, the GelMA/GelMA-AEMA blend promoted a more stable cartilage-like phenotype over a 21-day in vitro study, as evidenced by stronger downregulation of COL1A1, upregulation of COL2A1, improved compressive properties, and a noticeably higher redifferentiation index. These findings suggest that the blend not only supports chondrogenic redifferentiation but also enhances the mechanical integrity of bioprinted constructs, which are crucial requirements for cartilage tissue engineering.

Overall, this work emphasizes the critical role of rheological tuning in bioink design and offers a new material platform that connects superior printability with biological performance. Future research envisages the expansion of these findings by exploring GelMA/GelMA-AEMA at different ratios and evaluating in vivo outcomes such as matrix formation and tissue remodeling in long-term studies to assess clinical translation potential in cartilage repair.

## 5. Materials and Methods

### 5.1. Solutions and Physical Hydrogels Preparation

Photocrosslinkable functionalized gelatin-based biopolymers, namely gelatin methacryloyl (GelMA) and gelatin methacryloyl-aminoethyl-methacrylate (GelMA-AEMA), were synthesized from bovine gelatin type B following the procedure described by Van Hoorick et al. (2017) [19], which typically results in GelMA with a high degree of functionalization. Although no ^1^H NMR was performed in this study, the synthesis was carried out under equivalent conditions and kindly provided to us by Dr. Van Hoorick. Polymer solutions were prepared by dissolving the biopolymers in phosphate buffered saline (PBS) solution (1X, pH 7.4) (Lonza, Bornem, Belgium) at 50 °C for 30 min, under mild magnetic stirring, to the nominal concentration of 10 *w*/*v*%. GelMA/GelMA-AEMA hydrogel blends were obtained by blending equal volume fractions of GelMA and GelMA-AEMA, both at the concentration of 10 *w*/*v*%.

### 5.2. Bulk Shear Rheology

All physical hydrogel specimens were prepared in situ using a stress-controlled Physica MCR501 shear rheometer with temperature control (Anton Paar GmbH, Graz, Austria), coupled with a solvent trap to minimize solvent evaporation. The set-up was maintained at 37 °C and 1 mL of each polymer solution was dispensed on the bottom plate of the rheometer. The top plate of 50 mm was lowered to a final gap of 0.5 mm and excess solution was removed. The temperature was set to 20 °C and gelation was allowed to occur under oscillatory loading with a strain amplitude of 1%, with the rheometer in controlled-shear rate mode and an angular frequency of 10 rad.s^−1^. The dynamic moduli were monitored over time until apparent steady-state conditions were reached, i.e., the moduli were not drastically increasing with time anymore, allowing for the subsequent measurements to be performed.

#### 5.2.1. Oscillatory Shear Measurements with and Without Encapsulated Human Periosteum-Derived Cells and Their Expansion Protocol

Temperature, strain- and stress-amplitude, and frequency sweep measurements were applied to physically gelled GelMA and GelMA/GelMA-AEMA at 20 °C immediately after in situ specimen preparation. The thermo-mechanical rheological behavior of the physical hydrogels was assessed via temperature sweep experiments at an angular frequency of 10 rad.s^−1^ and a strain level of 1% from 20 to 40 °C at a ramp rate of +1 °C.min^−1^. Strain-amplitude sweep experiments were performed at an angular frequency of 10 rad.s^−1^ at strain levels ranging from 0.1 to 10,000% while monitoring the dynamic moduli of cell-free GelMA and the GelMA/GelMA-AEMA blend. Stress-amplitude sweep experiments were performed at an angular frequency of 10 rad.s^−1^ at shear stresses ranging from 5 to 5000 Pa under controlled shear stress mode while monitoring the dynamic moduli of cell-free GelMA 10 *w*/*v*% and cell-laden GelMA 10 *w*/*v*% in presence of human periosteum-derived cells (hPDCs). This stress-driven approach was chosen, as higher cell densities incorporated to GelMA were hypothesized to drastically change the viscoelastic behavior of the system; therefore, the stress-amplitude test would directly provide a measure of yield stress within the broad applied range of stresses [53]. Frequency sweep experiments were implemented either at a strain amplitude of 1% or at a stress-amplitude of 10 Pa and angular frequencies extending from 0.1 to 100 rad.s^−1^, while monitoring the dynamic moduli within the LVE. Cell-free GelMA 10 *w*/*v*% and the GelMA/GelMA-AEMA hydrogel blend were studied at a strain-amplitude of 1%, whereas cell-free and cell-laden GelMA 10 *w*/*v*% were subjected to a stress-amplitude of 10 Pa. The aim of the experiment was to verify the influence of the cells in the mechanical spectrum of GelMA at the nominal cell densities of 1, 10, and 20 million cells per milliliter. Each measurement was executed at least in triplicate and, for strain-amplitude and frequency sweep measurements, 8 data points per strain, stress, or frequency decade were acquired. All results comply with torque levels of at least one order of magnitude greater than the minimum torque limit in oscillatory mode, which for the Physica MCR 501 corresponds to 0.01 μN.m.

Human periosteum-derived cells (hPDC) were isolated from periosteal biopsies of different donors undergoing distraction osteogenesis surgeries, as previously described [54]. A sampling size *n* = 4 composed of 2 male and 2 female donors at the age of 13 ± 2.74 (M ± SD) years old was used. Briefly, the periosteum was stripped from the tibia with a periosteal lifter and specimens were maintained in growth medium. Subsequently, the tissue was minced and digested overnight at 37 °C in type IV collagenase (440 units/mg) (Invitrogen, Merelbeke, Belgium) in Dulbecco’s Modified Eagle medium (DMEM, Invitrogen, Merelbeke, Belgium), supplemented with 10% fetal bovine serum (FBS, BioWhittaker, Verviers, Belgium) and antibiotic–antimycotic solution (100 units/mL penicillin, 100 μg.mL^−1^ streptomycin, and 0.25 μg.mL^−1^ amphotericin B; Invitrogen, Merelbeke, Belgium). The resultant periosteal cells were collected by centrifugation and seeded in a T-25 flask (Thermo Scientific, Waltham, MA, USA) in growth medium. The hPDC pools were expanded until passage 9 in vitro at 37 °C, 5% CO_2_, and 95% humidity in T175 flasks (Thermo Scientific, USA), in presence of Dulbecco’s Modified Eagle medium (DMEM, Invitrogen, Merelbeke, Belgium), supplemented with 10% fetal bovine serum (FBS, BioWhittaker, Verviers, Belgium) and 1% antibiotic–antimycotic solution (100 units mL^−1^ penicillin, 100 mg mL^−1^ streptomycin, and 0.25 mg.mL^−1^ amphotericin B; Invitrogen, Merelbeke, Belgium). Medium was changed every 2–3 days, and cells were harvested with TrypLE Express (Life Technologies, Cramlington, UK) at a confluence of 80–90%. TrypLE Express was used for all passaging and harvesting steps during cell handling. hPDCs were chosen to evaluate the effect of cell densities of 1, 10, and 20 million cells per milliliter in the linear viscoelastic properties of GelMA. These cells represent a more cost-effective yet relevant option than the induced pluripotent stem cells used in the actual biological performance assessment of the materials.

#### 5.2.2. Shear Creep and Recovery Measurements and Determination of the Parameters of the Burgers Viscoelastic Model

Creep and creep-recovery tests were conducted within the linear viscoelastic regime at a constant stress of 50 Pa and stress removal, respectively, to GelMA 10 *w*/*v*% and GelMA/GelMA-AEMA specimens without cells, while measuring the resulting shear strain as a function of time. This was chosen because creep measurements within the linear regime (linear creep) at a constant temperature can provide information on the microstructures of materials and their time-dependent mechanical behavior. For both creep and creep-recovery, data points were acquired every 1 s. Stress was applied over 3600 s in order to assure stationary flow behavior. The materials were equally allowed to recover for 3600 s immediately after stress removal. Confirmation of terminal flow was given by comparing the magnitudes of the viscous term of the stationary region of the creep curve with the steady-state compliance, as the magnitude of the former must be at least as large as the one of the latter 176. The steady-state creep compliances of the creep and recovery stages were also compared as an extra measure to confirm terminal flow. All the tests were conducted at the temperature of 20 °C. The creep data were analyzed and represented in terms of the shear strain as a function of time.

The mechanical model selected to represent the creep curve of GelMA and GelMA/GelMA-AEMA hydrogels was the Burgers’ viscoelastic model. This model describes the creep behavior of four-element viscoelastic liquids and consists of a Maxwell element (viscoelastic liquid) connected in series with a Kelvin–Voigt element (viscoelastic solid). The retardation time of the Burgers model, as in Costa et al. (2015) [55], was adjusted to the shear creep data using exponential regression through the damped least-squares method (Levenberg–Marquardt algorithm). The goodness of the adjustment was given by the adjusted coefficient of determination (Adj. R-squared) using Origin, Version 2018b (OriginLab Corporation, Northampton, MA, USA). The creep curve of a Burgers’ fluid is given by Equation (1).

(1) $$\gamma \left(t\right)=\tau_{0}\left[\frac{1}{G_{M}}+\frac{1}{\eta_{M}}t+\frac{1}{G_{K}}\left(1-e^{-\frac{t}{\lambda }}\right)\right],$$

where *τ*_0_ is the preset shear stress, and *G*, *η*, and *λ* are the elastic constant, the viscosity, and the retardation time, correspondingly. The indices M and K refer to the Maxwell and Kelvin–Voigt elements, respectively.

#### 5.2.3. Steady-State Rotational Shear Measurements and Determination of the Herschel–Bulkley Model Parameters

Rotational shear measurements were implemented within the range of shear rates, extending from 0.1 to 100 s^−1^ for the GelMA/GelMA-AEMA hydrogel blend. The Herschel–Bulkley model was adjusted to the flow curve data of the hydrogel blend. This is a generalized model of non-Newtonian fluids [41] given by Equation (2), which is suitable for materials showing a yield point followed by pseudoplastic behavior.

(2) $$\tau \left(\dot{\gamma }\right)=\tau_{0}+m{\dot{\gamma }}^{n},$$

where *τ*_0_ is the yield stress (Pa), *m* (Pa.sn) is the consistency index, and *n* is the flow index. Data points were recorded at a steady-state condition and 10 data points per shear rate decade were acquired. The Herschel–Bulkley model was adjusted to the flow curve data points using the damped least-squares method (Levenberg–Marquardt algorithm) and the goodness of the adjustment was given by the adjusted coefficient of determination (Adj. R-squared) using Origin, Version 2018b (OriginLab Corporation, Northampton, MA, USA).

As suggested by Jiang et al. (2019) [39], the Von Mises yield criterion can be used to estimate a maximum attainable theoretical height of the printed construct. This can be achieved based on a balance between the calculated value of yield stress and gravitational forces, as those are the primary forces acting on the material at rest after extrusion. The stacking of material layers above a certain threshold can cause yielding at the infinitesimal thin film of the bottommost filament, which withstands all the mass above it. Hence, the maximum theoretical height, *h*, of the construct, in millimeters, above which yielding is susceptible to occur, is given by Equation (3).

(3) $$h\geq \frac{\sqrt{3}}{10^{3}}\left(\frac{\tau_{0}}{\rho g}\right),$$

where ρ is the density of the hydrogel, taken as 1000 kg.m^−3^, g is the gravitational acceleration constant, and τ_0_ is the yield stress (Pa) as determined from the Herschel–Bulkley model. This estimation does not consider detrimental effects related to instrumental errors of 3D-printing devices and their resolutions.

### 5.3. 3D-Printing Validation

Three-dimensional (3D) printing was performed to validate the new photocrosslinkable gelatin-based hydrogel blend under processing conditions via extrusion 3D printing. The procedure was carried out to investigate its ability to form filaments and fabricate 3D structures in comparison with those of the gold-standard GelMA as a benchmark. The BioCAD (regenHU, Villaz-Saint-Pierre, Switzerland) software was utilized in the design of the 3D virtual models and obtention of their G-code spatial coordinates. The 3D model consisted of squares of 10 mm in length by 10 mm in width, and a layer height corresponding to 80% of the nominal inner diameter of the printing nozzle. The experiment was implemented using a 3DDiscovery BioSafety 3D bioprinting system (regenHU, Villaz-Saint-Pierre, Switzerland). Materials were dispensed via a piston-driven dispensing printhead from a glass syringe (GasTight, Hamilton Company, Reno, NV, USA) through a SmoothFlow™ 25-gauge tapered nozzle (Nordson EFD, Zaventem, Belgium), equivalent to a nominal inner diameter of 0.250 mm. The temperatures of the cartridge and printing platform were set to 20 °C and controlled by the cooling bath accessory of the 3D bioprinting device throughout the process via a feedback loop from temperature sensors located at the printhead and printing platform. The temperature of the printing chamber was room temperature, corresponding to 20 ± 2 °C. Based on a visual observation of onset of stable filament formation, the flow rates of GelMA and GelMA-AEMA were set to 3 and 1 μL.s^−1^, respectively. Initially, GelMA and GelMA/GelMA-AEMA were 3D printed at the printing speed of 10 mm.s^−1^ to highlight the differences in superficial aspect between the two materials. Next, an optimal printing speed for GelMA/GelMA-AEMA was estimated using Equation (4). This equation was derived by assuming the printing speed (*v_P_*), which is the speed of the collector in millimeters per second, as the average fluid velocity exiting the nozzle during dispensing and the application of macroscopic mass conservation. The aim of this estimation was to validate the new hydrogel blend under optimal printing conditions.

(4) $$v_{P}\approx \frac{4}{\pi }\frac{Q}{d^{2}},$$

where *Q* is the volumetric flow rate in microliters per second, and *d* is the inner diameter of the nozzle tip, given in millimeters.

Two layers of each material were deposited onto regular microscopy glass slides as printing substrates. Imaging of the 3D-printed structures at room temperature (20 ± 2 °C) was obtained using a digital microscope coupled to a computer. Brightness and contrast corrections were performed using the Fiji flavor of the ImageJ software, version 1.53c [56].

### 5.4. Biological Evaluation of Encapsulated iPSC-Derived Chondrocytes

#### 5.4.1. Induced Pluripotent Stem Cells Expansion and Chondrogenic Differentiation

Human iPSC-derived chondrocytes were obtained by dissociating iPSC-derived cartilage nodules that were formed as previously described [57]. Briefly, CY2 human-induced pluripotent stem cells (hiPSCs, Rutgers University Cell and DNA Repository) were expanded on mitomycin C (Sigma)-treated SNL feeder cells in human embryonic stem cell (hESC) medium, consisting of Dulbecco’s Modified Eagle medium F12 (DMEM-F12, Gibco, Waltham, MA, USA) and supplemented with 1% sodium pyruvate, 20% knockout serum replacement (Invitrogen, ThermoFisher, Merelbeke, Belgium), 2 mM Glutamax (Gibco, USA), 1% non-essential amino acids (NEAA, Gibco, USA), 0.1 mM β-mercaptoethanol (Sigma-Aldrich, Overijse, Belgium), 50 U and 50 mg/mL penicillin/streptomycin (Pen/Strep, Invitrogen, ThermoFisher, Merelbeke, Belgium), and 10 ng/mL human basic fibroblast growth factor (hbFGF, PeproTech, London, UK). Culture medium was refreshed daily and hiPSCs were passaged weekly. When a sufficient cell count was reached, iPSCs were transferred to Matrigel-coated well plates and cultured in Essential 8 medium (Gibco, USA). Mesoderm induction was performed by culturing the hiPSCs for 36 h in Stemdif APEL medium (STEMCELL Technologies, Vancouver, BC, Canada), supplemented with 8 µM CHIR99021 (GSK3β inhibitor, Axon Medchem, Groningen, Netherlands), 50 U and 50 mg/mL Pen/Strep, and 20 ng/mL hbFGF, followed by another 36 h in the same APEL medium supplemented with 50 U and 50 mg/mL Pen/Strep, 8 ng/mL hbFGF, and 1 µM retinoic acid. Thereafter, chondrogenic differentiation was induced. In short, the cells were cultured in DMEM supplemented with 1% fetal bovine serum (FBS, BioWest, Nuaillé, France), 50 U and 50 mg/mL Pen/Strep, 1% L-glutamine, 1% NEAA, 1% sodium pyruvate, 1% insulin-transferrin-selenite X (ITS-X), 50 µg/mL ascorbic acid (AA, Sigma-Aldrich, Overijse, Belgium), 0.1 mM β-mercaptoethanol, 10 ng/mL TGF-β1, 10 ng/mL BMP-2, 10 ng/mL GDF-5, and 10 ng/mL hbFGF for 2 weeks to form cartilage-like nodules. The nodules were then detached and cultured in suspension for 7 weeks in the aforementioned medium without hbFGF.

#### 5.4.2. Hydrogel Precursor Preparation and Fabrication of Chondrocyte-Laden Hydrogel Constructs

Hydrogel precursor solutions were prepared by dissolving GelMA or a 1:1 blend of GelMA and GelMA-AEMA in PBS (20% *w*/*v*) at 40 °C and adding lithium phenyl-2,4,6-trimethylbenzoylphosphinate (LAP, CAS 85073-19-4) photoinitiator (6 mM).

Dissociation of the iPSC-derived cartilage nodules into single cells was performed by a two-step procedure including 25 min incubation in 2 mg mL^−1^ pronase (≈7.0 U mg^−1^, Roche, CH) in PBS + 1% antibiotic/antimycotic solution (100x) (AA, Gibco, USA) at 37 °C whilst rotating, followed by incubation in 1.5 mg mL^−1^ collagenase B (>0.15 U mg^−1^, Roche, Basel, Switzerland) in Dulbecco’s Modified Eagle’s medium (DMEM)-F12 (Gibco, USA) + 1% AA until full dissociation. Both enzyme solutions were filtered (0.22 µm) before use. Resulting cell suspension was passed through a 70 µM cell strainer to remove debris, centrifuged, and resuspended in PBS. Human iPSC-derived chondrocytes in PBS were resuspended to the hydrogel precursor solutions, resulting in bioinks with final concentration of 10% (*w*/*v*) and cell density of 2.10^7^ cells/mL. In order to obtain cell-laden samples with a homogeneous structure and a levelled surface, the bioinks were then pipetted into custom-made PTFE molds (adapted from Loessner et al. [58]) with 50 × 4 × 2 mm cavities, covered with glass slides, and crosslinked in a UV crosslinker (8W, UVP-1000) at 365 nm for 10 min. The resulting strips were divided into 4 × 4 × 2 mm constructs using a sterile scalpel and a cutting guide. As positive controls, micro-masses of iPSC-derived chondrocytes were prepared by seeding 37 µL droplets of a 2.10^7^ cells/mL solution in Nunc Cell Culture Treated Plates (Thermo Fisher, Waltham, MA, USA) and allowing precipitation for 3 h at 37 °C, 5% CO_2_, and 95% RH.

#### 5.4.3. In Vitro Culture

Cell-laden constructs and micro-masses were cultured in individual wells of a 24-well suspension well plate (Cellstar, Greiner, Germany). All samples were then cultured in a chemically defined, xeno-free chondrogenic medium [45] consisting of low-glucose DMEM (Gibco, USA), supplemented with 1 mM ascorbate-2-phosphate (Sigma-Aldrich, Belgium), 100 nM dexamethasone (Sigma, Belgium), 40 μg/mL proline (Sigma, Belgium), and ITS+ Premix Universal Culture Supplement (BD Biosciences, San Jose, CA, USA). This differentiation medium contained 10 ng/mL TGF-β1, 0.2 ng/mL fibroblast growth factor 2 (FGF-2, R&D systems), 100 ng/mL BMP-2 (Inductos, Belgium), 1 ng/mL BMP-6 (PeproTech, UK), and 100 ng/mL GDF-5 (PeproTech, UK). Negative control samples were cultured in DMEM-C, consisting of DMEM supplemented with 10% FBS and 1% AA. Samples were cultured for up to 21 days at 37 °C, 5% CO_2_, and 95% RH, and the medium was refreshed every 2 days.

#### 5.4.4. Cell Viability

To assess cell viability, GelMA/GelMA-AEMA blend samples (*n* = 3) were analyzed on day 1, 4, and 7 after construct fabrication with the LIVE/DEAD^TM^ viability/cytotoxicity kit (Invitrogen, USA) according to the manufacturer’s protocol. In brief, samples were washed twice in phosphate buffered saline (PBS, Lonza, Swiss Alps), stained with LIVE/DEAD staining solution (0.5 µL calcein-AM and 2 µL Ethidium-Homodimer-1 per mL PBS) for 30 min at 37 °C, 5% CO_2_, and 95% RH. After another two washes in PBS, the samples were imaged with an inverted microscope (Olympus IX83) and CellSens version 4.3.1 software (Olympus, Hamburg, Germany).

#### 5.4.5. Cell-Laden Construct Homogenization

Cell-laden constructs and micro-mass controls (*n* = 3) were washed twice in PBS, snap frozen in liquid nitrogen on day 1, 14, and 21 of in vitro culture, and stored at −80 °C until analysis. Frozen samples were then submerged in lysis buffer consisting of buffer RLT (Qiagen, Hilden, Germany) with 1% (*v*/*v*) β-mercaptoethanol and mechanically homogenized (Precellys, Bertin Instuments, France) in lysis tubes (CK28-R, Bertin Instruments, Montigny le Bretonneux, France) for 3 cycles of 15 s with 10 s pauses in between. The resulting lysate was centrifuged (10 min 24900 RCF) before use.

#### 5.4.6. DNA Quantification

Cell-laden construct lysates were diluted 1:10 in ddH_2_O and measured with a Quant-it DNA Assay kit (Invitrogen) according to the manufacturer’s protocol. Briefly, 15 µL of diluted lysate was added to 195 µL of working solution (working buffer + 1:200 chromogen) and the colorimetric read-out was compared to 2 standards. The resulting concentration was multiplied by 10 to account for the dilution and multiplied by the lysate volume to reach the absolute DNA quantity per sample.

#### 5.4.7. RNA Extraction and Gene Expression Analysis

RNA extraction was performed on undiluted lysate of the cell-laden constructs and micro-masses using a RNeasy mini kit (Qiagen, Germany), according to manufacturer’s protocol. Total RNA was quantified with a Nanodrop 2000 system (Qiagen, Germany) and copyDNA (cDNA) was synthesized, starting from 500 ng of RNA per sample, using a PrimeScript RT reagent kit (TakaraBio, Kusatsu, Japan). The resulting cDNA was diluted to 5 ng/mL and used for quantitative PCR with an intercalating dye (TB Green Premix Ex Taq II, TakaraBio, Japan) using a Rotor-gene machine (Corbett Research RG-6000, UK) and incubated as follows: 10 s at 95 °C, 15 s at 60 °C, and 20 s at 72 °C. The primer sequences for the investigated genes can be found in the Supplementary Information, Table S1.

#### 5.4.8. Histology and Immunohistochemistry

On day 1, 14, and 21 of in vitro culture, cell-laden gel constructs and micro-masses (*n* = 3) were washed twice in PBS, fixed in 4% paraformaldehyde and washed in PBS again. Fixed samples were dehydrated through ethanol series of 50, 70, and 90% and embedded in paraffin. Microtome sections of 5 µm were made in the horizontal as well as vertical direction prior to staining. The sections were deparaffinized in Histoclear (National Diagnostics, France), dehydrated in methanol, and rehydrated in DI water before staining with 0.5% Alcian Blue 8GX solution (Sigma, Belgium) for 30 min. After washing in DI water, a 0.1% nuclear Fast Red (Clin-Tech, Rotherham, UK) counterstaining for the nuclei was performed for 5 min. The sections were washed in DI water and dehydrated through ethanol series, cleared in Histoclear, and mounted with Pertex mounting medium (Histolab, Gothenburg, Sweden). Immunological stainings to detect human collagen type I and type II were performed on deparaffinized and dehydrated samples. Enzymatic antigen retrieval was performed for 15 min in 1 mg/mL pepsin (Sigma-Aldrich, Belgium) in 0.02 M HCl; thereafter, PBS with 0.1% Tween20 (PBS-T) was used as a washing buffer. To block endogenous peroxidase activities, quenching with 3% H_2_O_2_ was performed twice for 5 min, followed by washing buffer. To prevent unspecific binding, a blocking step with 5% bovine serum albumin (BSA) with PBS-T + 0.01% Triton X100 (blocking buffer) was applied for 30 min before adding the primary antibodies. Next, anti-collagen type I antibody (1:200 dilution, PA1-36057, Thermo Fisher, USA) or anti-collagen type II antibody (1:20 dilution, AB761, Merck-Millipore, USA) in blocking buffer was applied for overnight incubation at 4 °C. Negative control samples with only blocking buffer (without primary antibody) were used as negative controls. After washing the samples and performing another blocking step, the secondary antibody, horseradish peroxidase (HRP)-conjugated goat anti-rabbit (dilution 1:500, 111-035-003, Jackson ImmunoResearch, UK) was applied for 30 min. After washing, peroxidase activity was visualized with 3,3′-Diaminobenzidine (DAB) (Enzo, Belgium). To stop the reaction, samples were washed in DI water, then counterstained with hematoxylin and rinsed with tap water. The slides were then dehydrated and mounted as mentioned above. All stainings were imaged with an inverted light microscope (Olympus IX83), using cellSens version 4.3.1 software (Olympus, Germany).

#### 5.4.9. Statistical Analyses of Biological Testing

DNA quantification was plotted as mean ± standard deviation with *n* = 3. An unpaired *t*-test with Welch correction per time point was performed and individual variances were computed for each comparison.

Gene expression analysis of iPSC-derived chondrocytes encapsulated in GelMA/GelMA-AEMA hydrogel blend and GelMA hydrogel were compared to cells only (micro-masses). The relative mRNA expression of cell-laden hydrogels was represented with the 2^−ΔΔCt^ method [20], with HPRT1 as the housekeeping gene, and was normalized to gel-free conditions (micro-masses) on day 1. In addition, the ratio of COL2A1 to COL1A1 expression of iPSC-derived chondrocytes encapsulated in GelMA/GelMA-AEMA blend and GelMA was compared to gel-free conditions (micro-mass). A 2-way ANOVA followed by Tukey’s multiple comparisons test was performed, with * *p* < 0.05, ** *p* < 0.01, *** *p* < 0.001, and *n* = 3.

### 5.5. Mechanical Properties of UV-Cured Constructs

Quasi-static compression testing was performed using a LM1 TestBench mechanical testing device (TA Instruments, New Castle, DE, USA), coupled with an in-house built frame mounted on a configurable base plate, allowing for vertical uniaxial compression, and a 2.5 N load cell with a precision of 0.5% of full scale. Hybrid hydrogel specimens with dimensions of 4 × 4 × 2 mm (W × L × D), prepared from GelMA and GelMA/gelMA-AEMA, as previously described, were tested. A maximum engineering strain of 20% was set to avoid influence of the bottom plate. The aim of the experiment was to characterize the evolution of the elastic moduli and number densities of active network chains of cell-free and cell-laden hydrogels under in vitro differentiation culturing, as previously described. Table 2 presents the composition of the specimens studied.

The deformation rate applied was 0.2 mm.s^−1^ and the tests were conducted at the temperature of 25 °C. The results were interpreted using the neo-Hookean model, which derives from the affine network theory, a fundamental molecular approach to rubber elasticity [59]. This model assumes that each network strand deforms affinely with the macroscopic deformation and that the network is ideal, neglecting defects such as dangling chains, loops, physical entanglements, and excluded volume interactions. In such a model, the relative deformation of each network strand is the same as the macroscopic relative deformation imposed on the whole network [59]. Actual networks are not perfect, and a significant number of defects are present [60]. Despite its simplicity, the neo-Hookean model provides a useful description of the entropic elasticity of polymer networks. The stress–stretch relationship under uniaxial compression, assuming incompressibility (Poisson’s ratio of 0.5), is described by Equation (5) [61].

(5) $$\sigma =K\left(\alpha -\frac{1}{\alpha^{2}}\right);\;\alpha =\frac{l_{z}}{L_{z}},$$

where *σ* is the engineering stress, *K* is the elastic modulus in compression, and *α* is the stretch ratio given by the ratio of the current sample length, *l_z_*, and the initial sample length, *L_z_*. Note that in compression, *α* < 1. Equation (5) describes a purely elastic, single-phase material response; however, in hydrogels, additional effects such as fluid pressurization arise due to their biphasic nature, consisting of fluid and solid phases. This becomes particularly relevant when loading rates exceed relaxation rates associated with interstitial fluid flow. The model was adjusted to the experimental data using the damped least-squares method (Levenberg–Marquardt algorithm) to estimate the modulus K, which is related to the number density of elastically active chains and the absolute temperature. The goodness of the adjustment is given by the adjusted coefficient of determination (Adj. R-squared). Calculations were performed using Origin, Version 2018b (OriginLab Corporation, Northampton, MA, USA). The data are presented in terms of mean values, and the error bars correspond to the standard deviation of the mean. All conditions were tested at least in quadruplicate and more detailed information can be found in Figure S7 and Tables S2 and S3 of the Supplementary Information. Statistical analyses using two-sample *t*-tests were performed to compare the mean values of the elastic modulus obtained from specimens in compression testing, as shown in Table 2. The *t*-tests did not assume equal variances. The analyses were computed using Origin, Version 2018b (OriginLab Corporation, Northampton, MA, USA) at a significance level of *p* < 0.01.

## Figures and Tables

**Table 1:** Burgers model parameters. The indices M and K refer to the Maxwell and Kelvin–Voigt elements, respectively.

Material	G_M_ (Pa)	G_K_ (Pa)	η_M_ (Pa.s)	η_K_ (Pa.s)	λ (s)
GelMA	650	1700	7 × 10^6^	1.2 × 10^6^	712
GelMA/GelMA-AEMA	90	100	5 × 10^5^	6.4 × 10^4^	644

**Table 2:** Sample size and compositions of specimens tested under compression testing.

Material	Culturing Period (Day)	Initial Cell Density (mL^−1^)	Sample Size, *n*
GelMA 10 *w*/*v*%	1	2 × 10^7^	5
		0	5
	21	2 × 10^7^	4
		0	5
GelMA/GelMA-AEMA 1:1, 10 *w*/*v*%	1	2 × 10^7^	5
		0	5
	21	2 × 10^7^	6
		0	6

## Data Availability

The datasets generated and analyzed during the present study are available from the corresponding author upon reasonable request. All relevant data are included within the manuscript and its Supplementary Information File. This study is available from the corresponding author on reasonable request.

## Supplementary Materials

The following supporting information can be downloaded at: https://www.mdpi.com/article/10.3390/gels11090698/s1, Figure S1: Adjustment of the creep stage data points to the Burgers’ model: (**a**) GelMA and (**b**) GelMA/GelMA-AEMA. Figure S2: Rheological monitoring of photocrosslinking reaction via the storage moduli of GelMA and GelMA/GelMA-AEMA in presence of 0.02 *w*/*v*% LAP under incidence of UV light (3500 mW, 365 nm) at an angular frequency of 10 rad.s^−1^, 1% strain, and 37 °C. Figure S3: Alcian Blue and staining for glycosaminoglycans (**top**) and immunohistochemistry for collagen type II (**bottom**) of iPSC-derived chondrocytes in gel-free micro-mass culture. Scale bar: 200 µm. Figure S4: Immunohistological staining anti-human collagen type I of iPSC-derivedchondrocyte-laden GelMA/GelMA-AEMA hydrogel blend, GelMA hydrogel, and gel-free micro-mass culture. Scale bar: 200 µm. Table S1: Primer sequences for qPCR. Abbreviations: HPRT (hypoxanthine-guanine phosphoribosyltransferase), COL1a1 (alpha-1 type I collagen), COL2a1 (alpha-1 type II collagen), COL10a1 (alpha-1 type X collagen), ACAN (aggrecan), and SOX9 (SRY-Box Transcription Factor). Figure S6: Alcian Blue staining for glycosaminoglycans in a cast (**top**) and bioprinted (**bottom**) construct of iPSC-derived chondrocytes in GelMA/GelMA-AEMA blends. Scale bar: 200 µm. Table S2: Summarized two-sample *t*-test comparison for GelMA/GelMA-AEMA and GelMA cell-laden constructs between day 1 and day 21 (*p* < 0.01). Table S3: Summarized statistics via two-sample *t*-test comparison for GelMA and GelMA/gelMA-AEMA cell-laden constructs at day 21 (*p* < 0.01). Figure S7: Engineering stress versus stretch ratio of specimens of GelMA at D1 (**a**) control and (**b**) in presence of cells; GelMA/GelMA-AEMA at D1 (**c**) control and (**d**) in presence of cells; GelMA at D21 (**e**) control and (**f**) in presence of cells; and GelMA-GelMA-AEMA at D21 (**g**) control and (**h**) in presence of cells.

## Abbreviations

The following abbreviations are used in this manuscript:

AA	Ascorbic acid
ACAN	Aggrecan
ANOVA	Analysis of variance
BMP	Bone morphogenetic protein
cDNA	Complementary DNA
COL10A1	Collagen type X alpha 1 chain
COL1A1	Collagen type I alpha 1 chain
COL2A1	Collagen type II alpha 1 chain
ddH_2_O	Double-distilled water
DMEM	Dulbecco’s modified eagle medium
DNA	Deoxyribonucleic acid
EBB	Extrusion-based 3D bioprinting
FBS	Fetal bovine serum
GDF-5	Growth differentiation factor 5
GelMA	Gelatin methacryloyl
GelMA-AEMA	Gelatin methacryloyl-aminoethyl-methacrylate
hbFGF	Human basic fibroblast growth factor
hESC	Human embryonic stem cells
hPDC	Human periosteum-derived cells
HPRT1	Hypoxanthine Phosphoribosyltransferase 1
HRP	Horseradish peroxidase
iPSC	Induced-pluripotent stem cell
LAP	Lithium phenyl-2,4,6-trimethylbenzoylphosphinate
LVR	Linear viscoelastic regime
mRNA	Messenger RNA
PBS	Phosphate buffered saline
PTFE	Polytetrafluoroethylene
RH	Relative humidity
RNA	Ribonucleic acid
RUNX2	Run-related transcription factor 2
SNL	STO-neo-leukemia
SOX9	Sex determining region Y—box 9
TE	Tissue engineering
TGF-β1	Transforming growth factor beta 1
UV	Ultraviolet light

## References

[1] Sohn S., Van Buskirk M., Buckenmeyer M.J., Londono R., Faulk D. (2020). Whole Organ Engineering: Approaches, Challenges, and Future Directions. Appl. Sci..

[2] Jaipan P., Nguyen A., Narayan R.J. (2017). Gelatin-Based Hydrogels for Biomedical Applications. MRS Commun..

[3] Klotz B.J., Gawlitta D., Rosenberg A.J.W.P., Malda J., Melchels F.P.W. (2016). Gelatin-Methacryloyl Hydrogels: Towards Biofabrication-Based Tissue Repair. Trends Biotechnol..

[4] Wang X., Ao Q., Tian X., Fan J., Tong H., Hou W., Bai S. (2017). Gelatin-Based Hydrogels for Organ 3D Bioprinting. Polymers.

[5] Ying G., Jiang N., Yu C., Zhang Y.S. (2018). Three-Dimensional Bioprinting of Gelatin Methacryloyl (GelMA). Biodes Manuf..

[6] Van Hoorick J., Tytgat L., Dobos A., Ottevaere H., Van Erps J., Thienpont H., Ovsianikov A., Dubruel P., Van Vlierberghe S. (2019). (Photo-)Crosslinkable Gelatin Derivatives for Biofabrication Applications. Acta Biomater..

[7] Moroni L., Boland T., Burdick J.A., De Maria C., Derby B., Forgacs G., Groll J., Li Q., Malda J., Mironov V.A. (2018). Biofabrication: A Guide to Technology and Terminology. Trends Biotechnol..

[8] Zhang B., Korolj A., Lai B.F.L., Radisic M. (2018). Advances in Organ-on-a-Chip Engineering. Nat. Rev. Mater..

[9] Schuurman W., Klein T.J., Dhert W.J.A., van Weeren P.R., Hutmacher D.W., Malda J. (2015). Cartilage Regeneration Using Zonal Chondrocyte Subpopulations: A Promising Approach or an Overcomplicated Strategy?. J. Tissue Eng. Regen. Med..

[10] Billiet T., Gevaert E., De Schryver T., Cornelissen M., Dubruel P. (2014). The 3D Printing of Gelatin Methacrylamide Cell-Laden Tissue-Engineered Constructs with High Cell Viability. Biomaterials.

[11] Yin J., Yan M., Wang Y., Fu J., Suo H. (2018). 3D Bioprinting of Low-Concentration Cell-Laden Gelatin Methacrylate (GelMA) Bioinks with a Two-Step Cross-Linking Strategy. ACS Appl. Mater. Interfaces.

[12] Townsend J.M., Beck E.C., Gehrke S.H., Berkland C.J., Detamore M.S. (2019). Flow Behavior Prior to Crosslinking: The Need for Precursor Rheology for Placement of Hydrogels in Medical Applications and for 3D Bioprinting. Prog. Polym. Sci..

[13] Mendoza-Cerezo L., Rodríguez-Rego J.M., Macías-García A., Callejas-Marín A., Sánchez-Guardado L., Marcos-Romero A.C. (2024). Three-Dimensional Bioprinting of GelMA Hydrogels with Culture Medium: Balancing Printability, Rheology and Cell Viability for Tissue Regeneration. Polymers.

[14] Billiet T., Vandenhaute M., Schelfhout J., Van Vlierberghe S., Dubruel P. (2012). A Review of Trends and Limitations in Hydrogel-Rapid Prototyping for Tissue Engineering. Biomaterials.

[15] Zhu M., Wang Y., Ferracci G., Zheng J., Cho N.J., Lee B.H. (2019). Gelatin Methacryloyl and Its Hydrogels with an Exceptional Degree of Controllability and Batch-to-Batch Consistency. Sci. Rep..

[16] Gaglio C.G., Baruffaldi D., Pirri C.F., Napione L., Frascella F. (2024). GelMA Synthesis and Sources Comparison for 3D Multimaterial Bioprinting. Front. Bioeng. Biotechnol..

[17] Sun T., Feng Z., He W., Li C., Han S., Li Z., Guo R. (2023). Novel 3D-Printing Bilayer GelMA-Based Hydrogel Containing BP, β-TCP and Exosomes for Cartilage–Bone Integrated Repair. Biofabrication.

[18] Pourazariyan A., Shahgholi M., Karimipour A. (2025). The Effect of Initial Temperature and ZnO Nanoparticle Volume Fractions on the Stability of Sodium Alginate Hydrogel Nanocomposite Using Molecular Dynamics Simulation. Int. Commun. Heat. Mass. Transf..

[19] Van Hoorick J., Gruber P., Markovic M., Tromayer M., Van Erps J., Thienpont H., Liska R., Ovsianikov A., Dubruel P., Van Vlierberghe S. (2017). Cross-Linkable Gelatins with Superior Mechanical Properties Through Carboxylic Acid Modification: Increasing the Two-Photon Polymerization Potential. Biomacromolecules.

[20] Jain T., Baker H.B., Gipsov A., Fisher J.P., Joy A., Kaplan D.S., Isayeva I. (2021). Impact of Cell Density on the Bioprinting of Gelatin Methacrylate (GelMA) Bioinks. Bioprinting.

[21] Majumder N., Mishra A., Ghosh S. (2022). Effect of Varying Cell Densities on the Rheological Properties of the Bioink. Bioprinting.

[22] Groot R.D., Bot A., Agterof W.G.M. (1996). Molecular Theory of Strain Hardening of a Polymer Gel: Application to Gelatin. J. Chem. Phys..

[23] Michon C., Chapuis C., Langendorff V., Boulenguer P., Cuvelier G. (2004). Strain-Hardening Properties of Physical Weak Gels of Biopolymers. Food Hydrocoll..

[24] Ferry J.D. (1980). Viscoelastic Properties of Polymers.

[25] Calafel M.I., Criado-Gonzalez M., Aguirresarobe R., Fernández M., Mijangos C. (2025). From Rheological Concepts to Additive Manufacturing Assessment of Hydrogel-Based Materials for Advanced Bioprinting Applications. Mater. Adv..

[26] Radi B., Wellard R.M., George G.A. (2013). Effect of Dangling Chains on the Structure and Physical Properties of a Tightly Crosslinked Poly(Ethylene Glycol) Network. Soft Matter.

[27] Amin D., Wang Z. (2020). Nonlinear Rheology and Dynamics of Supramolecular Polymer Networks Formed by Associative Telechelic Chains under Shear and Extensional Flows. J. Rheol..

[28] Kokol V., Pottathara Y.B., Mihelčič M., Perše L.S. (2021). Rheological Properties of Gelatine Hydrogels Affected by Flow- and Horizontally-Induced Cooling Rates during 3D Cryo-Printing. Colloids Surf. A Physicochem. Eng. Asp..

[29] Elharfaoui N., Djabourov M., Babel W. (2007). Molecular Weight Influence on Gelatin Gels: Structure, Enthalpy and Rheology. Macromol. Symp..

[30] Djabourov M., Boccara N., Daoud M. (1985). Gelation of Physical Gels: The Gelatin Gels. Physics of Finely Divided Matter.

[31] Ross-Murphy S.B. (1992). Structure and Rheology of Gelatin Gels: Recent Progress. Polymer.

[32] Picout D.R., Ross-Murphy S.B. (2003). Rheology of Biopolymer Solutions and Gels. Sci. World J..

[33] Yang Z., Hemar Y., Hilliou L., Gilbert E.P., McGillivray D.J., Williams M.A.K., Chaieb S. (2016). Nonlinear Behavior of Gelatin Networks Reveals a Hierarchical Structure. Biomacromolecules.

[34] Mouser V.H.M., Melchels F.P.W., Visser J., Dhert W.J.A., Gawlitta D., Malda J. (2016). Yield Stress Determines Bioprintability of Hydrogels Based on Gelatin-Methacryloyl and Gellan Gum for Cartilage Bioprinting. Biofabrication.

[35] Sánchez-Sánchez R., Rodríguez-Rego J.M., Macías-García A., Mendoza-Cerezo L., Díaz-Parralejo A. (2023). Relationship between Shear-Thinning Rheological Properties of Bioinks and Bioprinting Parameters. Int. J. Bioprint.

[36] Liu W., Heinrich M.A., Zhou Y., Akpek A., Hu N., Liu X., Guan X., Zhong Z., Jin X., Khademhosseini A. (2017). Extrusion Bioprinting of Shear-Thinning Gelatin Methacryloyl Bioinks. Adv. Healthc. Mater..

[37] Wilson S.A., Cross L.M., Peak C.W., Gaharwar A.K. (2017). Shear-Thinning and Thermo-Reversible Nanoengineered Inks for 3D Bioprinting. ACS Appl. Mater. Interfaces.

[38] Smith P.T., Basu A., Saha A., Nelson A. (2018). Chemical Modification and Printability of Shear-Thinning Hydrogel Inks for Direct-Write 3D Printing. Polymer.

[39] Jiang T., Munguia-Lopez J.G., Flores-Torres S., Kort-Mascort J., Kinsella J.M. (2019). Extrusion Bioprinting of Soft Materials: An Emerging Technique for Biological Model Fabrication. Appl. Phys. Rev..

[40] Du D., Sugita N., Liu Z., Moriguchi Y., Nakata K., Myoui A., Yoshikawa H. (2015). Repairing Osteochondral Defects of Critical Size Using Multiple Costal Grafts: An Experimental Study. Cartilage.

[41] Morrison F.A. (2001). Understanding Rheology.

[42] Levato R., Webb W.R., Otto I.A., Mensinga A., Zhang Y., van Rijen M., van Weeren R., Khan I.M., Malda J. (2017). The Bio in the Ink: Cartilage Regeneration with Bioprintable Hydrogels and Articular Cartilage-Derived Progenitor Cells. Acta Biomater..

[43] Messaoudi O., Henrionnet C., Bourge K., Loeuille D., Gillet P., Pinzano A. (2020). Stem Cells and Extrusion 3D Printing for Hyaline Cartilage Engineering. Cells.

[44] Visser J., Gawlitta D., Benders K.E., Toma S.M., Pouran B., Ren van Weeren P., Dhert W.J., Malda J. (2015). Endochondral Bone Formation in Gelatin Methacrylamide Hydrogel with Embedded Cartilage-Derived Matrix Particles. Biomaterials.

[45] Pirosa A., Gottardi R., Alexander P.G., Puppi D., Chiellini F., Tuan R.S. (2021). An in Vitro Chondro-Osteo-Vascular Triphasic Model of the Osteochondral Complex. Biomaterials.

[46] Li X., Chen S., Li J., Wang X., Zhang J., Kawazoe N., Chen G. (2016). 3D Culture of Chondrocytes in Gelatin Hydrogels with Different Stiffness. Polymers.

[47] Loeser R. (2009). Aging and Osteoarthritis: The Role of Chondrocyte Senescence and Aging Changes in the Cartilage Matrix. Osteoarthr. Cartil..

[48] Castro-Viñuelas R., Sanjurjo-Rodríguez C., Piñeiro-Ramil M., Hermida-Gómez T., Fuentes-Boquete I.M., de Toro-Santos F.J., Blanco-García F.J., Díaz-Prado S.M. (2018). Induced Pluripotent Stem Cells for Cartilage Repair: Current Status and Future Perspectives. Eur. Cell Mater..

[49] Lee J., Taylor S.E.B., Smeriglio P., Lai J., Maloney W.J., Yang F., Bhutani N. (2015). Early Induction of a Prechondrogenic Population Allows Efficient Generation of Stable Chondrocytes from Human Induced Pluripotent Stem Cells. FASEB J..

[50] Agten H., Van Hoven I., Viseu S.R., Van Hoorick J., Van Vlierberghe S., Luyten F.P., Bloemen V. (2022). In Vitro and in Vivo Evaluation of 3D Constructs Engineered with Human IPSC-Derived Chondrocytes in Gelatin Methacryloyl Hydrogel. Biotechnol. Bioeng..

[51] Bachmann B., Spitz S., Schädl B., Teuschl A.H., Redl H., Nürnberger S., Ertl P. (2020). Stiffness Matters: Fine-Tuned Hydrogel Elasticity Alters Chondrogenic Redifferentiation. Front. Bioeng. Biotechnol..

[52] Schuh E., Hofmann S., Stok K., Notbohm H., Müller R., Rotter N. (2012). Chondrocyte Redifferentiation in 3D: The Effect of Adhesion Site Density and Substrate Elasticity. J. Biomed. Mater. Res. A.

[53] Amorim P.A., d’Ávila M.A., Anand R., Moldenaers P., Van Puyvelde P., Bloemen V. (2021). Insights on Shear Rheology of Inks for Extrusion-Based 3D Bioprinting. Bioprinting.

[54] Eyckmans J., Roberts S.J., Schrooten J., Luyten F.P. (2010). A Clinically Relevant Model of Osteoinduction: A Process Requiring Calcium Phosphate and BMP/Wnt Signalling. J. Cell Mol. Med..

[55] Costa I., Barros J. (2015). Tensile Creep of a Structural Epoxy Adhesive: Experimental and Analytical Characterization. Int. J. Adhes. Adhes..

[56] Schindelin J., Arganda-Carreras I., Frise E., Kaynig V., Longair M., Pietzsch T., Preibisch S., Rueden C., Saalfeld S., Schmid B. (2012). Fiji: An Open-Source Platform for Biological-Image Analysis. Nat. Methods.

[57] Yamashita A., Morioka M., Yahara Y., Okada M., Kobayashi T., Kuriyama S., Matsuda S., Tsumaki N. (2015). Generation of Scaffoldless Hyaline Cartilaginous Tissue from Human IPSCs. Stem Cell Rep..

[58] Loessner D., Meinert C., Kaemmerer E., Martine L.C., Yue K., Levett P.A., Klein T.J., Melchels F.P.W., Khademhosseini A., Hutmacher D.W. (2016). Functionalization, Preparation and Use of Cell-Laden Gelatin Methacryloyl-Based Hydrogels as Modular Tissue Culture Platforms. Nat. Protoc..

[59] Rubinstein M., Colby R.H. (2003). Polymer Physics.

[60] Toda M., Morita H. (2018). Rubber Elasticity of Realizable Ideal Networks. AIP Adv..

[61] Sperling L.H. (2005). Introduction to Physical Polymer Science.

