# Supplementary material for: Novel GelMA/GelMA-AEMA Hydrogel Blend with Enhanced Printability as a Carrier for iPSC-Derived Chondrocytes In Vitro
Source: Gels. 2025 Sep 2;11(9):698. doi: 10.3390/gels11090698 (PMC12469456; doi:10.3390/gels11090698)
Supplement: Supplementary file 1 [file gels-11-00698-s001.zip › gels-3782655-supplementary.pdf]

gels

MDPI

Supplementary information

Adjustment of the Burgers viscoelastic model

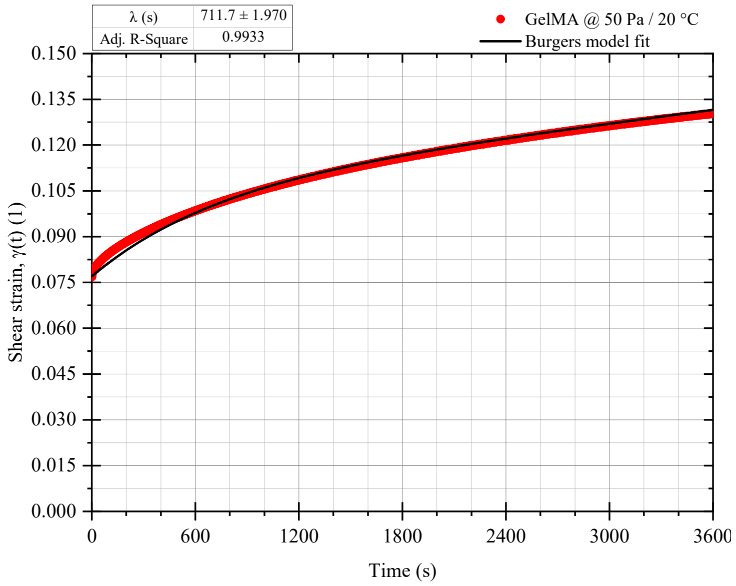

(a)

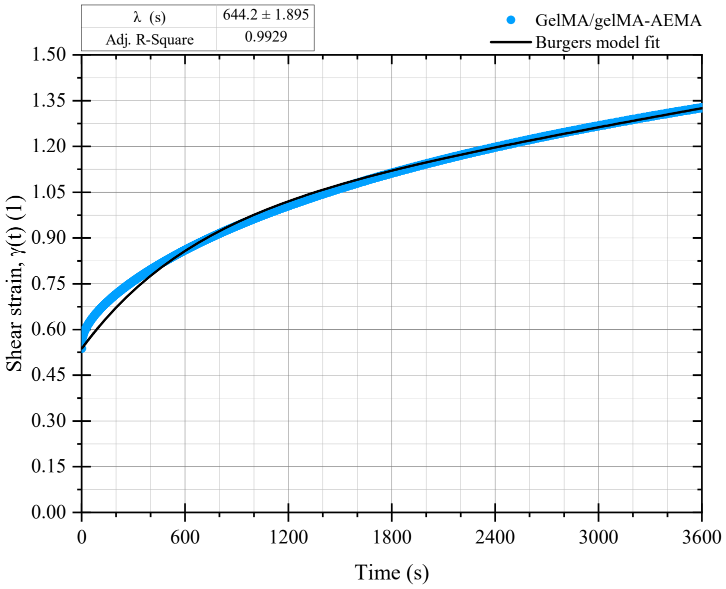

(b)

Figure S1. Adjustment of the creep stage data points to the Burgers' model (a) GelMA (b) GelMA/GelMA-AEMA.

Photocrosslinking measurements under UV incidence via shear rheology

Methods

GelMA and GelMA-AEMA solutions were prepared and mixed as described in the main text. Specimens of GelMA and GelMA/GelMA-AEMA containing 0.02 w/v% of the photoinitiator lithium phenyl-2,4,6-trimethylbenzoylphosphinate (LAP) (CAS 85073-19-4, Sigma-Aldrich, Overijse, Belgium) were prepared *in situ* from the sol state using a stress-controlled Physica MCR 301 parallel plate shear rheometer with temperature control (Anton Paar GmbH, Graz, Austria). The rheometer was coupled with an accessory

solvent trap to minimize solvent evaporation. The set-up was maintained at 37 °C, and 300 μL of each polymer solution was dispensed on the bottom plate of the rheometer. The top plate of 25 mm was lowered to a final gap of 0.3 mm and excess solution was removed. The chemical crosslinking reaction was allowed to occur at 37 °C under UV incidence (3500 mW, 365 nm, EXFO Novacure 2000). The storage modulus, G′, of the materials was monitored for 300 s via small-amplitude oscillatory shear rheometry at an engineering strain amplitude of 1% and an angular frequency of 10 $rad.s^{-1}$. Each measurement was executed in triplicate and one data point was acquired every second. All the reported results agree to torque levels of at least one order of magnitude greater than the minimum torque limit of the device in oscillatory mode, which for the Physica MCR 301 corresponds to 0.01 μN.m. The mesh size of the chemical gels, r_{mesh}, which is proportional to the average chain length between crosslinks and was estimated assuming that mean-field theory is held[52], is given by the expression in Equation S1.

$$r_{mesh} \approx \left(\frac{6RT}{\pi N_{AV} G'}\right)^{\frac{1}{3}} \qquad \text{Eq. S1}$$

Where R = 8.31 $m^3.Pa.K^{-1}.mol^{-1}$ is the universal gas constant, T is the absolute temperature, N_{AV} = 6.02×10^{23} mol^{-1} is the Avogadro's number and G′ is the storage modulus.

Results

Small-amplitude oscillatory shear rheology was used to monitor the evolution of the UV-mediated crosslinking reaction, leading to chemical and permanent network formation for GelMA and GelMA/GelMA-AEMA in the presence of 0.02 w/v% LAP. The storage modulus (G′) over time, with UV irradiation starting at t_0 = 0, is presented in Figure S2. Both chemical hydrogels converged to nearly the same value of G′ after 300 seconds of UV exposure, despite the GelMA/GelMA-AEMA blend having a higher crosslinking potential due to its increased number of reactive sites. At a plateau level of approximately 3 kPa, both materials presented similar mesh sizes, estimated to be around 14 nm. This suggests that the crosslinking potential of the GelMA/GelMA-AEMA blend has been likely limited by the photoinitiator concentration used in this experiment. Additionally, this result reflects the relaxed state prior to swelling in culturing conditions.

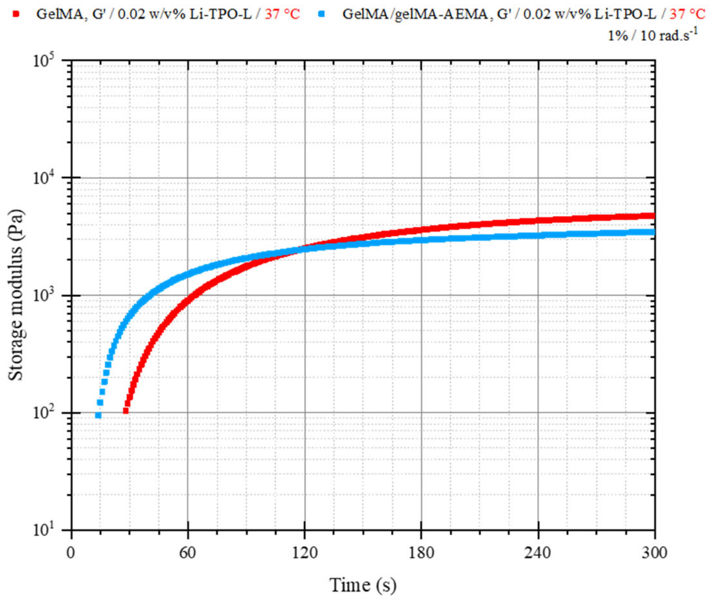

Figure S2. Rheological monitoring of photocrosslinking reaction via the storage moduli of GelMA and GelMA/GelMA-AEMA in presence of 0.02 w/v% LAP under incidence of UV light (3500 mW, 365 nm) at an angular frequency of 10 rad.s^{-1}, 1% strain and 37 °C.

Histology and immunohistochemistry

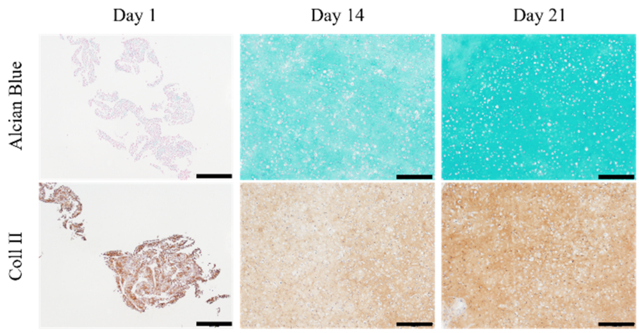

Figure S3. Alcian Blue and staining for glycosaminoglycans (top) and immunohistochemistry for collagen type II (bottom) of iPSC-derived chondrocytes in gel-free micro-mass culture. Scale bar: 200 µm.

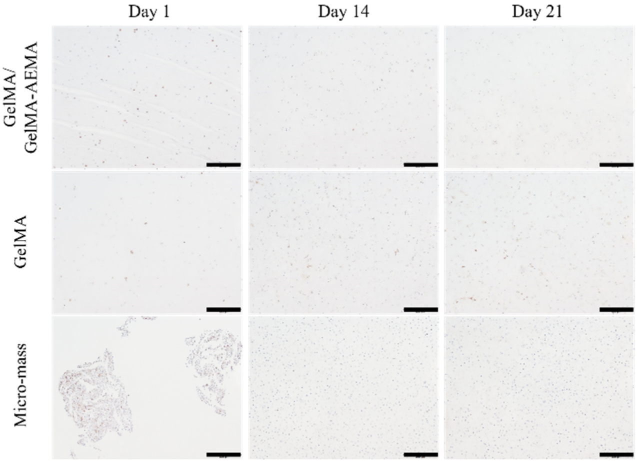

Figure S4. Immunohistological staining anti-human collagen type I of iPSC-derivedchondrocyte-laden GelMA/GelMA-AEMA hydrogel blend, GelMA hydrogel and gel-free micro-mass culture. Scale bar: 200 µm.

Table S1. Primer sequences for qPCR. Abbreviations: HPRT (hypoxanthine-guanine phosphoribosyltransferase), COL1a1 (alpha-1 type I collagen), COL2a1 (alpha-1 type II collagen), COL10a1 (alpha-1 type X collagen), ACAN (aggrecan), SOX9 (SRY-Box Transcription Factor).

GENE	FORWARD (5’ 3’)	REVERSE (5’ 3’)
**HPRT**	TGAGGATTTGGAAAGGGTGT	GAGCACACAGAGGGCTACAA
**COL1A1**	GACGAAGACATCCCACCAAT	AGATCACGTCATCGCACAAC
**COL2A1**	GGCTTCCATTTCAGCTATGG	AGCTGCTTCGTCCAGATAGG
**COL10A1**	ACGATACCAAATGCCCACAG	GTGGACCAGGAGTACCTTGC
**ACAN**	GTCTCACTGCCCAACTAC	GGAACACGATGCCTTTCAC
**SOX9**	TGGAGACTTCTGAACGAGAGC	CGTTCTTCACCGACTTCCTC
**RUNX2**	CGCATTCCTCATCCCAGTAT	GCCTGGGGTCTGTAATCTGA

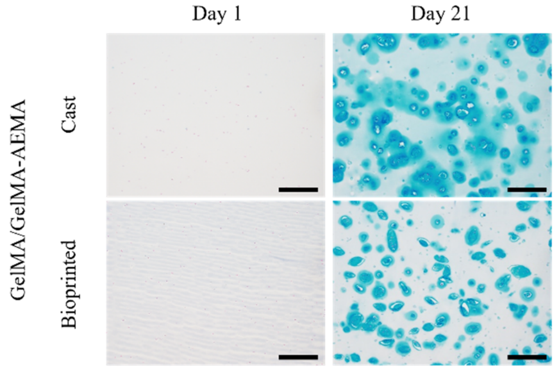

Figure S6. Alcian Blue staining for glycosaminoglycans in a cast (top) and bioprinted (bottom) construct of iPSC-derived chondrocytes in GelMA/GelMA-AEMA blends. Scale bar: 200 µm.

Mechanical properties of UV cured constructs

Table S2. Summarized two-sample *t*-test comparison for GelMA/GelMA-AEMA and GelMA cell-laden constructs between day 1 and day 21 ($p < 0.01$).

Material	Time point (day)	n	Mean K (Pa)	SD (Pa)	*t*	DF	Prob. > |t|
GelMA/GelMA-AEMA	1	4	2010.84	9.99	148.76	5.00	2.59×10^{-10}
	21	6	67,687.55	1081.36			
GelMA	1	5	2334.72	10.48	106.59	4.00	4.62×10^{-10}
	21	5	41,050.61	812.11			

DF: degree of freedom

Table S3. Summarized statistics via two-sample *t*-test comparison for GelMA and GelMA/gelMA-AEMA cell-laden constructs at day 21 ($p < 0.01$).

Material	n	Mean K (Pa)	SD (Pa)	*t*	DF	Prob. > |t|
GelMA	5	41,050.61	812.11	46.59	8.94	5.55×10^{-12}
GelMA/GelMA-AEMA	6	67,687.55	1081.36			

DF: degree of freedom

Adjustment of the statistical rubber elasticity equation (Eq. V) to the data of compression testing of GelMA and GelMA/GelMA-AEMA constructs.

(a) Control n = 5

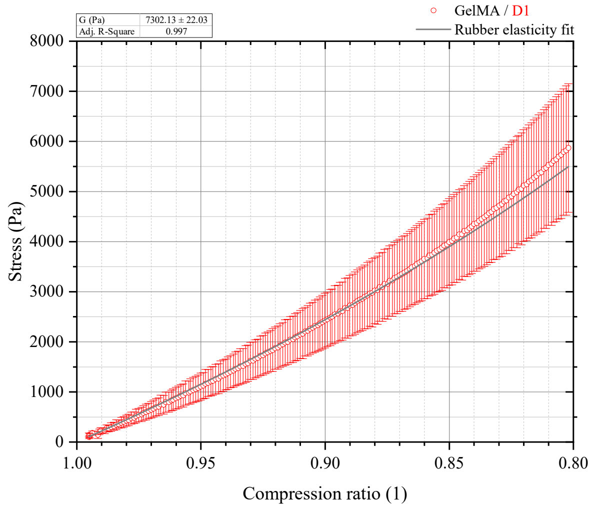

(b) n = 5

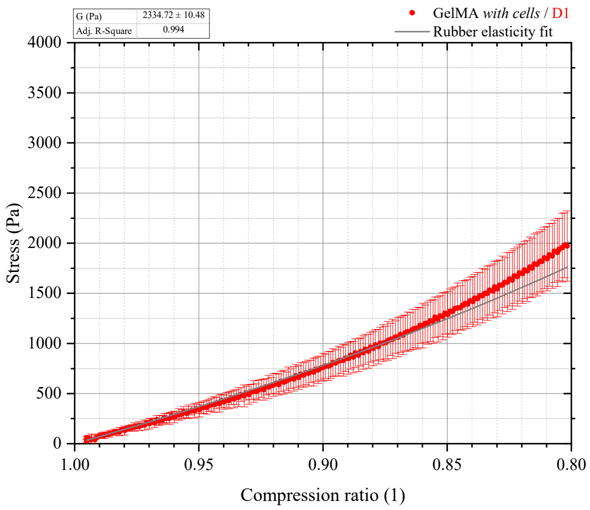

(c) Control, n = 5

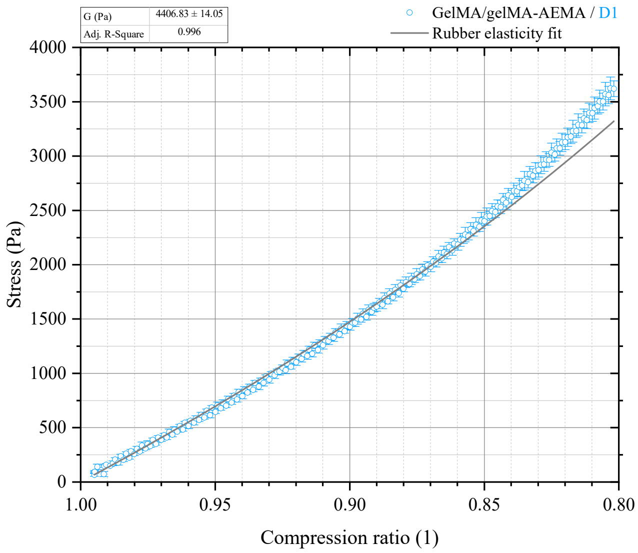

(d) n = 4

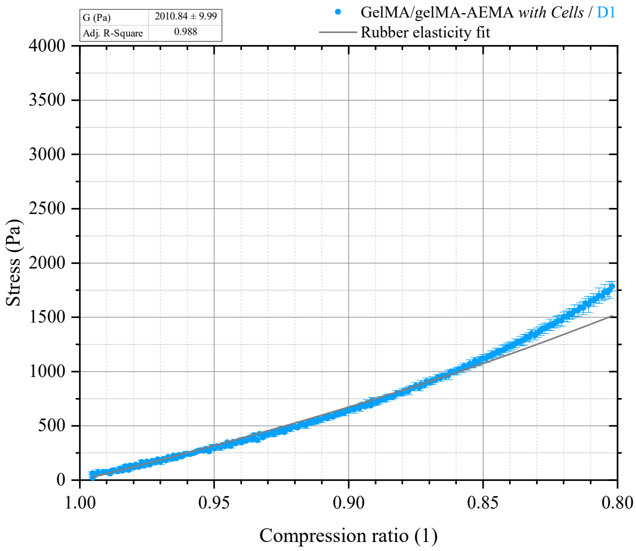

(e) Control, n = 5

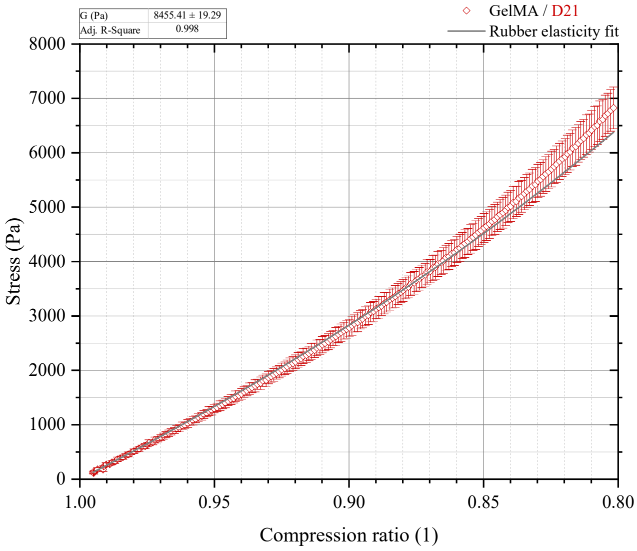

(f) n = 5

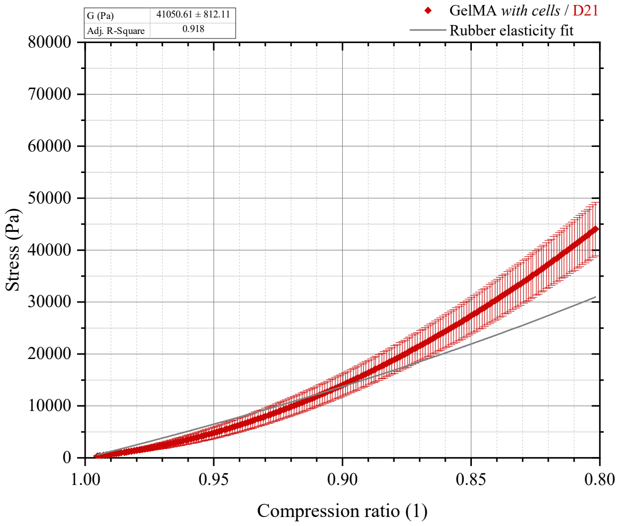

(g) Control, n = 6

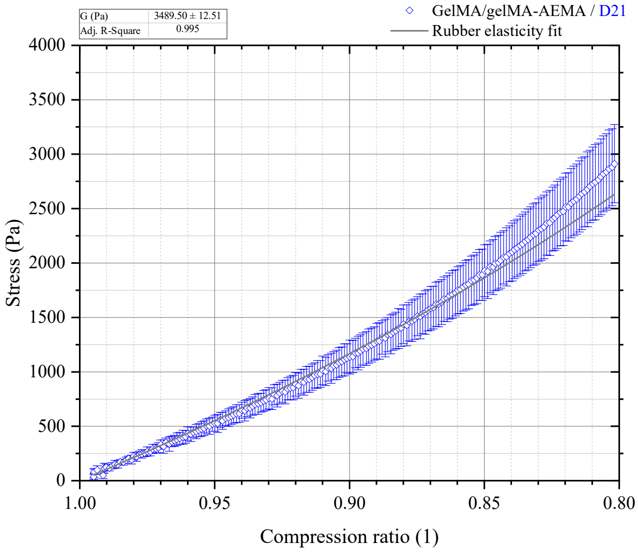

(h) n = 6

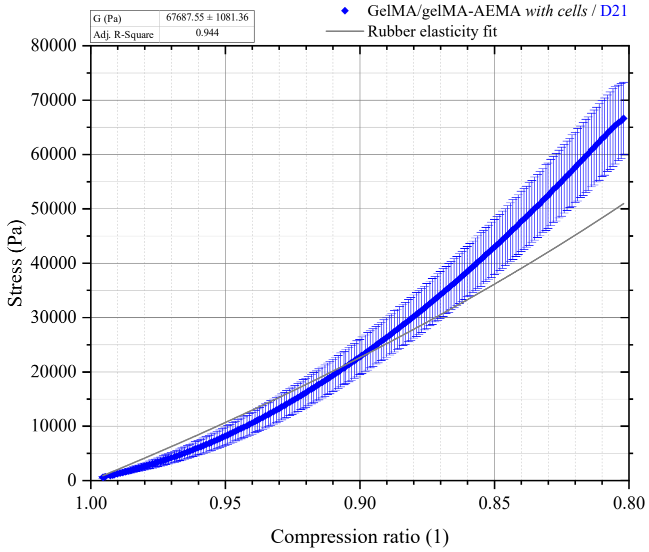

Figure S7. Engineering stress versus compression ratio of specimens of GelMA at D1 (a) control and (b) in presence of cells; GelMA/GelMA-AEMA at D1 (c) control and (d) in presence of cells; GelMA at D21 (e) control and (f) in presence of cells; GelMA-GelMA-AEMA at D21 (g) control and (h) in presence of cells.
